# Melatonin protects against cadmium-induced oxidative stress via mitochondrial STAT3 signaling in human prostate stromal cells

**DOI:** 10.1038/s42003-023-04533-7

**Published:** 2023-02-08

**Authors:** Moonjung Hyun, Hyejin Kim, Jehein Kim, Juhong Lee, Ho Jeong Lee, Laxmi Rathor, Jeremy Meier, Andrew Larner, Seon Min Lee, Yeongyu Moon, Jungil Choi, Sung Min Han, Jeong-Doo Heo

**Affiliations:** 1 Gyeongnam Biohealth Research Center, Gyeongnam Branch Institute, Korea Institute of Toxicology, Jinju, 52834 Republic of Korea; 2 Department of Physiology and Aging, College of Medicine, Institute on Aging, University of Florida, Gainesville, FL USA; 3 Division of Hematology, UNC School of Medicine, University of North Carolina, Chapel Hill, NC USA; 4 Department of Biochemistry and Molecular Biology, Virginia Commonwealth University, Richmond, VA USA

**Keywords:** Stress signalling, Toxicology

## Abstract

Melatonin protects against Cadmium (Cd)-induced toxicity, a ubiquitous environmental toxicant that causes adverse health effects by increasing reactive oxygen species (ROS) production and mitochondrial dysfunction. However, the underlying mechanism remains unclear. Here, we demonstrate that Cd exposure reduces the levels of mitochondrially-localized signal transducer and activator of transcription 3 (mitoSTAT3) using human prostate stromal cells and mouse embryonic fibroblasts. Melatonin enhances mitoSTAT3 abundance following Cd exposure, which is required to attenuate ROS damage, mitochondrial dysfunction, and cell death caused by Cd exposure. Moreover, melatonin increases mitochondrial levels of GRIM-19, an electron transport chain component that mediates STAT3 import into mitochondria, which are downregulated by Cd. In vivo, melatonin reverses the reduced size of mouse prostate tissue and levels of mitoSTAT3 and GRIM-19 induced by Cd exposure. Together, these data suggest that melatonin regulates mitoSTAT3 function to prevent Cd-induced cytotoxicity and could preserve mitochondrial function during Cd-induced stress.

## Abstract (web summary)

Melatonin protects against cadmium through regulation of mitoSTAT3 and reduction of ROS damage in human prostate stromal cells.

## Introduction

Melatonin (N-acetyl-5-methoxytryptamine) is a lipophilic hormone secreted by the pineal gland that can cross cell membranes and enter intracellular organelles^1,2^. In addition to its well-known function in regulating circadian rhythms, melatonin plays a critical role in preventing mitochondrial dysfunction by regulating the homeostasis of mitochondrial reactive oxygen species (mitoROS)^3–5^. Melatonin has also been shown to modulate cell death pathways, including apoptosis^6^ and autophagy^7^. Notably, melatonin has been reported to ameliorate oxidative stress and mitochondrial damage caused by cadmium (Cd) exposure in the plant, fish, and rat models^8–13^.

Cd is a naturally occurring toxic material that is found widely in food products, cigarette smoke, and industrial sources. Due to its very long biological half-life of 10-30 years, both acute and long-term Cd exposures have been associated with adverse health effects, including cardiovascular disease, liver disease, kidney disease, and prostate cancer^14–19^. Cd exposure has been reported to trigger apoptosis in various cell types^19–24^. Although Cd itself cannot directly form reactive oxygen species (ROS), increasing evidence has suggested that Cd exposure indirectly triggers various free radicals, triggering cell death and other adverse health effects^25–32^. In addition, studies have shown that Cd can directly damage isolated mitochondria^33^, suggesting that Cd-induced oxidative stress and mitochondrial dysfunction could underlie cell death after Cd exposure. However, the molecular mechanisms underlying the beneficial effects of melatonin against Cd-induced oxidative stress and mitochondrial damage are poorly understood.

Signal transducer and activator of transcription 3 (STAT3) plays key roles in organism development, stem cell proliferation, chronic inflammation, autoimmunity, energy metabolism, and cancer progression^34–38^. It was previously thought that STAT3 exerted these effects by acting as a nuclear transcription factor. However, accumulating evidence has indicated that mitochondrially-localized STAT3 (mitoSTAT3) also plays an important role in regulating the electron transport chain (ETC), mitochondrial DNA transcription, ATP production, cell death, and ROS homeostasis^34,36,38,39^.

In this study, we investigated the mechanism underlying the protective role of melatonin against Cd-toxicity in two mammalian cell lines including WPMY-1 human prostate stromal cells and mouse embryonic fibroblasts (MEFs). We selected WPMY-1 cells because Cd exposure has been associated with prostate cancer^14,19^. On the other hand, several studies reported evidence of Cd-induced apoptosis in cultured prostate cells^19,24^. We also used MEF because it is a well-established system in toxicology research and in evaluating the dynamic regulation of mitoSTAT3 in response to various cellular inputs, including oxidative stress and cytokines^40^. We demonstrated that Cd exposure decreases mitoSTAT3 levels to trigger mitochondrial dysfunction. Furthermore, we found that melatonin protects against Cd exposure by blocking the loss of mitoSTAT3, thereby maintaining mitochondrial integrity. Together, our findings suggest that melatonin regulates mitoSTAT3 function, thereby protecting against Cd-induced oxidative stress.

## Results

### Melatonin prevents Cd-induced cell death and mitochondrial dysfunction

**Fig. 1:**
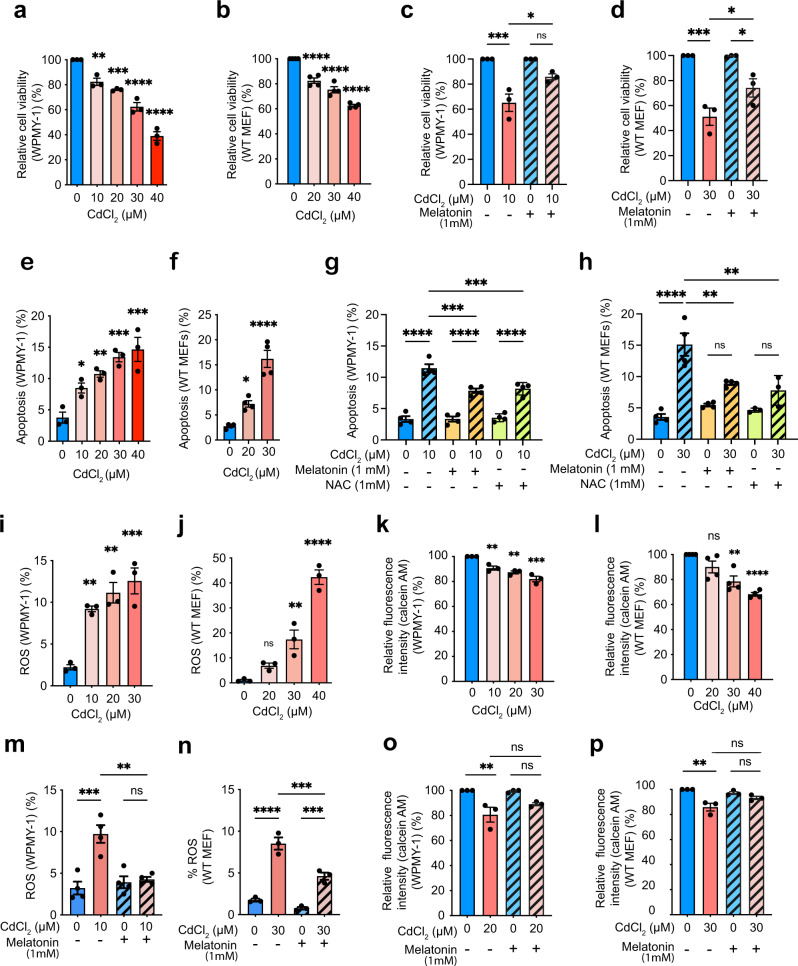
Melatonin pretreatment affects the decreased cell viability and mitochondrial dysfunction induced by Cd. **a**, **b** Cell viability after exposure to Cd for 24 h. **c**, **d** Cell viability after 1 h pretreatment with 1 mM melatonin before Cd exposure for 24 h. Apoptosis accessed by annexin-v/PI assay in WPMY-1s (**e**) and MEFs (**f**) after Cd exposure. Apoptosis of WPMY-1 (**g**) and MEF cells (**h**) pretreated with melatonin or NAC before Cd exposure.  mitoROS after Cd exposure in WPMY-1s (**i**) and MEFs (**j**). mPTP opening after Cd exposure in WPMY-1s (**k**) and MEFs (**l**). mitoROS production after melatonin pretreatment and Cd exposure in WPMY-1s (**m**) and MEFs (**n**) cells. mPTP opening after melatonin pretreatment and Cd exposure in WPMY-1s (**o**) and MEFs (**p**). All data were normalized to corresponding untreated controls. Data represent the mean ± SEM of at least 3–4 independent assays. **p < 0.005, ***p < 0.001, ****p < 0.0001; one-way ANOVA with Tukey’s post hoc test.

We exposed two independent cell lines (human prostate stromal cells [WPMY-1s] and mouse embryonic fibroblasts [MEFs]) to increasing CdCl_2_ concentrations over 24 h. The final CdCl_2_ concentration ranging from 10 to 40 μM was selected based on a previous manuscript reporting that the CdCl_2_ concentration significantly affecting cell viability is 30 μM, and the concentration resulting in 50% of cell viability is 68.6 μM in human airway epithelial cells^41^. This also reflects the range of Cd concentrations detected in human tissues^42^. The MTT cell viability assay showed that CdCl_2_ exposure reduced the viability of WPMY-1 cells (Fig. 1a) and MEFs (Fig. 1b) in a dose-dependent manner^43^. Previous studies have demonstrated that treating a pharmacological concentration of melatonin (1 mM) protects against various toxicants and cancer conditions^44–46^. Notably, pretreatment of cells with 1 mM melatonin for 1 h before exposure to CdCl_2_ increased the viability of CdCl_2_-exposed WPMY-1 cells (Fig. 1c) and MEFs (Fig. 1d) compared to the respective untreated control cells. In addition, exposure to CdCl_2_ increased apoptosis in a concentration- (Fig. 1e, f) and duration-dependent manner (Supplementary Fig. 1a, b). Melatonin pretreatment suppressed the increased apoptosis levels by CdCl_2_ exposure in both WPMY-1 cells (Fig. 1g) and MEFs (Fig. 1h). Together, these results indicate that melatonin treatment ameliorates cell death induced by CdCl_2_ exposure.

Previous studies have shown that Cd toxicity is associated with ROS and mitochondrial stress^7,47,48^. We evaluated mitoROS levels by staining cells using MitoSOX and analyzing their fluorescence intensity 24 h after CdCl_2_ treatment^49^. Exposure to CdCl_2_ markedly increased mitoROS levels in a dose-dependent manner (Fig. 1i, j). Oxidative stress can decrease mitochondrial membrane potential (MtMP) and promote the opening of the mPTP, an inner membrane protein complex that forms a non-specific channel which further increases ROS production and cell death^50–53^. We, therefore, evaluated the degree of mPTP opening after CdCl_2_ exposure using a calcein fluorescence-quenching assay^54^. Calcein fluorescence was significantly reduced in CdCl_2_-exposed WPMY-1 cells (Fig. 1k) and MEFs (Fig. 1l) in a concentration-dependent manner, indicating that CdCl_2_ exposure increases mPTP opening. Further, exposure to CdCl_2_ in WPMY-1 cells and MEFs reduced the intensity of TMRE staining, suggesting reduced mitochondrial ETC activity (Supplementary Fig. 1c, d). Melatonin pretreatment did not alter mitoROS levels under basal conditions without CdCl_2_ exposure but suppressed increased mitoROS levels in CdCl_2_-exposed cells compared to those treated without melatonin (Fig. 1m, n). In addition, melatonin pretreatment prevented the increase in mPTP and the decrease in mitochondrial membrane potential (MtMP) by CdCl_2_ exposure in both WPMY-1 (Fig.1o and Supplementary Fig. 1c) and MEF cells (Fig. 1p and Supplementary Fig. 1d). To evaluate whether the antioxidant function of melatonin is important for the protective effects against Cd exposure, we pretreated cells with NAC, a well-known antioxidant^55^. Similar to melatonin, the pretreatment of NAC before CdCl_2_ exposure suppressed the changes in apoptosis (Fig. 1g, h), MtMP (Supplementary Fig. 1c, d), and mitoROS (Supplementary Fig. 1e, f) in both cell lines. Together, these results indicate that melatonin can alleviate the adverse effects of Cd on cell viability and mitochondrial homeostasis, including increased mitoROS and mPTP opening, as well as decreased MtMP.

### Melatonin requires STAT3 to protect against Cd-induced toxicity

**Fig. 2:**
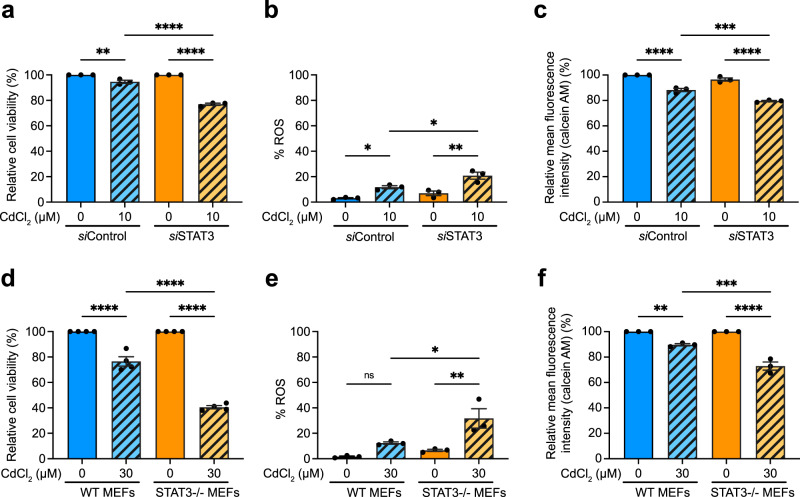
Effect of STAT3 deficiency on susceptibility to Cd toxicity. **a** Viability of WPMY-1 cells transfected with *si*Ctrl or *si*STAT3. **b** mitoROS levels in WPMY-1 cells exposed to Cd for 24 h after siRNA transfection. **c** mPTP opening in WPMY-1 cells exposed to Cd for 24 h after siRNA transfection. **d** Viability of WT MEFs and STAT3^–/–^ MEFs. **e** mitoROS levels in MEFs exposed to Cd for 24 h. **f** mPTP opening in WT and STAT3^–/–^ MEF cells exposed to Cd for 24 h. All data were normalized to corresponding untreated controls. Data represent the mean ± SEM of at least 3–4 independent assays. *p < 0.05, **p < 0.005, ***p < 0.001, ****p < 0.0001; one-way ANOVA with Tukey’s post hoc test.

STAT3 is associated with mitochondrial ETC activity, cellular respiration, ATP production, ROS generation, and mPTP opening^56,57^. Cells lacking STAT3 are more sensitive to oxidative stress^58^. We inhibited STAT3 expression by transfecting WPMY-1 cells with siRNA targeting STAT3 (*si*STAT3) before being exposed to CdCl_2_ for 24 h (Supplementary Fig. 1g). This condition did not alter cell viability under basal conditions without CdCl_2_ exposure. However, exposure to 10 µM CdCl_2_ significantly reduced the viability of *si*STAT3-transfected WPMY-1 cells compared to the non-targeting control siRNA (*si*Control)-transfected group (Fig. 2a and Supplementary Fig. 1h). Apoptosis assays also showed that *si*STAT3 cells have significantly increased apoptosis compared to the *si*Control group in response to 10 µM CdCl_2_ exposure (Supplementary Fig. 1a). *si*STAT3-cells also showed increased mitoROS (Fig. 2b), enhanced mPTP opening (Fig. 2c), and decreased MtMP compared to the *si*Control group (Supplementary Fig. 1c). Stat3-knockout MEFs (STAT3^–/–^ MEFs) also exhibited a stronger response to 30 µM CdCl_2_ exposure than wild-type (WT) MEFs, including a decrease in cell viability (Fig. 2d and Supplementary Fig. 1i) and the increases in apoptosis (Supplementary Fig. 1b), mitoROS production (Fig. 2e), and mPTP opening (Fig. 2f). The MtMP in Stat3-knockout MEFs was lower than that in WT MEFs under the basal condition, and CdCl_2_ exposure did not further decrease it (Supplementary Fig. 1d).

**Fig. 3:**
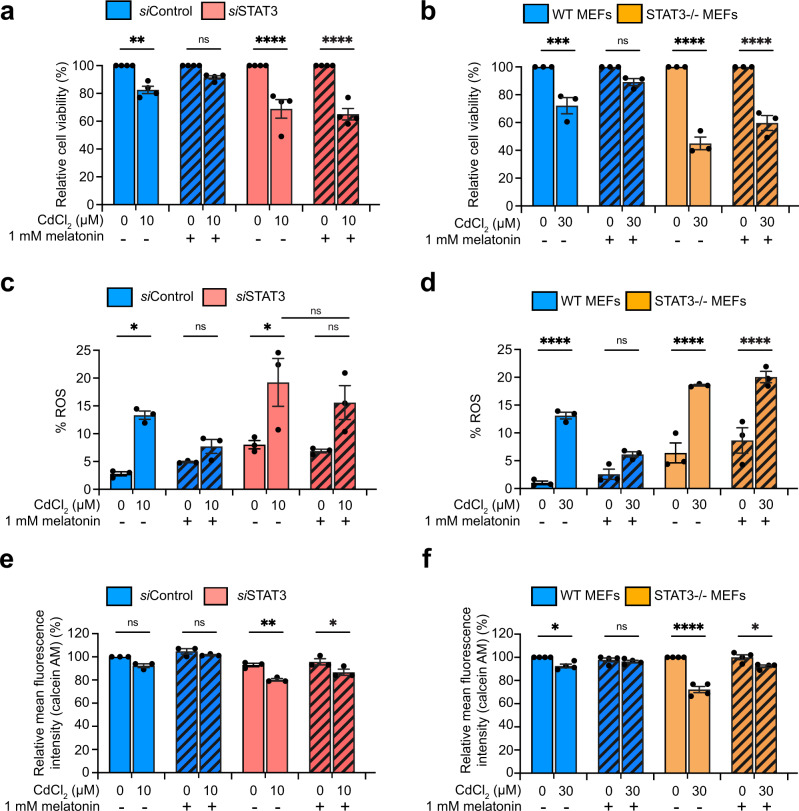
Effect of melatonin on STAT3-deficient WPMY-1 and MEF cells exposed to CdCl_2_. Cell viability of *si*STAT3-treated WPMY-1 cells (**a**) and STAT3^–/–^ MEFs (**b**) after melatonin pretreatment and Cd exposure for 24 h. mitoROS production in *si*STAT3-treated WPMY-1 cells (**c**) and STAT3^–/–^ MEFs (**d**) after melatonin pretreatment and Cd exposure for 24 h. mPTP opening in *si*STAT3-treated WPMY-1 cells (**e**) and STAT3^–/–^ MEFs (**f**). All data were normalized to corresponding untreated controls. Data represent the mean ± SEM of at least 3–4 independent assays. *p < 0.05, **p < 0.005, ***p < 0.001, ****p < 0.0001; one-way ANOVA with Tukey’s post hoc test.

Next, we determined whether melatonin acts through STAT3 to suppress the adverse effects of CdCl_2_ exposure. STAT3 deficiency suppressed the ability of melatonin to attenuate the harmful effects of CdCl_2_ on cell viability (Fig. 3a, b and Supplementary Fig. 2a, b), mitoROS (Fig. 3d), mPTP opening (Fig. 3e, f), and MtMP (Supplementary Fig. 1c, d). The only exception was mitoROS in *si*STAT3 WPMY-1 cells, in which melatonin pretreatment failed to significantly suppress the enhanced mitoROS level by CdCl_2_ exposure (Fig. 3c). However, this condition still showed a somewhat decreased ability of melatonin to reduce the mitoROS against CdCl_2_ exposure compared to *si*Control (Fig. 3c). These results indicate that STAT3 is required for melatonin to prevent CdCl_2_-induced mitochondrial dysfunction and cell death.

### Melatonin increases mitoSTAT3 to protect against Cd exposure

**Fig. 4:**
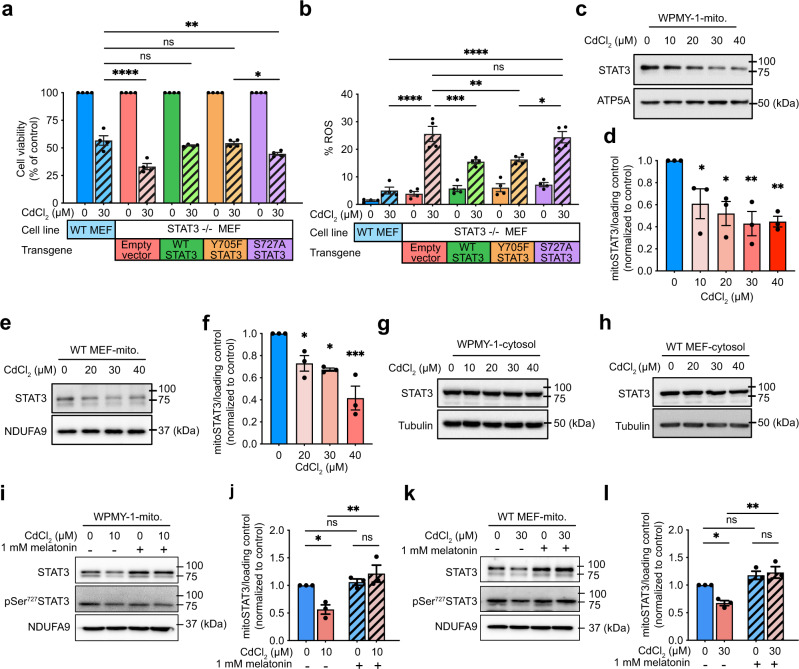
Melatonin restores mitoSTAT3 levels decreased by Cd exposure. Cell viability (**a**) and mitoROS levels (**b**) in WT MEFs, STAT3^–/–^ MEFs, or STAT3^–/–^ MEFs reconstituted with STAT3 WT or STAT3 Y705F, or STAT3 S727A and treated with CdCl_2_(c). **c** Representative immunoblot of mitoSTAT3 expression in WPMY-1 cells treated with CdCl_2_ for 24 h at the indicated concentrations. **d** Quantification of results from (**c**). STAT3 expression was normalized to the mitochondrial loading control, ATP5A. **e** Representative immunoblot of mitoSTAT3 expression in MEFs treated with CdCl_2_ for 24 h at the indicated concentrations. **f** Quantification of the results from (**e**). STAT3 expression was normalized to the mitochondrial loading control, NDUFA9. Representative immunoblots of STAT3 expression in the cytosolic extracts of WPMY-1 (**g**) and MEF (**h**) cells treated with CdCl_2_ for 24 h at the indicated concentrations. **i**–**l** STAT3 expression in mitochondrial extracts of cells treated with 1 mM melatonin for 1 h and CdCl_2_ for 24 h. **i** Representative immunoblot of mitoSTAT3 and pSer727 STAT3 in WPMY-1 cells. **j** Quantification of the results in panel i. STAT3 expression was normalized to the mitochondrial loading control, NDUFA9. **k** Representative immunoblot of mitoSTAT3 and pSer727 STAT3 in WT MEF cells. **l** Quantification of the results in panel k. STAT3 expression was normalized to the mitochondrial loading control, NDUFA9. Data represent the mean ± SEM of at least 3–4 independent assays. *p < 0.05, **p < 0.005, ***p < 0.001, ****p < 0.0001; one-way ANOVA with Tukey’s post hoc test.

To determine whether melatonin requires nuclear STAT3 or mitoSTAT3 to protect against Cd exposure, we performed functional rescue assays by overexpressing different STAT3 variants in STAT3^−/−^ MEFs (Supplementary Fig. 3a). WT-STAT3 overexpression enhanced the viability of STAT3^−/−^ MEFs exposed to CdCl_2_ to a similar degree as in WT MEFs and also partially suppressed increased mitoROS levels (Fig. 4a, b). pTyr^705^ induces the nuclear translocation of cytoplasmic STAT3, and subsequent pSer^727^ enhances the transcriptional activity of STAT3^59^. pSer^727^ also triggers STAT3’s mitochondrial translocation^34,36,40,59–61^. Expressing mutant Y705F-STAT3 suppressed the decreased cell viability and the increased mitoROS levels by CdCl_2_ exposure to a similar extent as WT-STAT3 (Fig. 4a, b). In contrast, S727A-STAT3 failed to rescue these to a similar level as WT STAT3 (Fig. 4a, b), suggesting that pSer^727^ in STAT3 is critical for protecting cells against Cd exposure.

Intrigued by these results, we further explored whether Cd exposure could affect the abundance of mitoSTAT3. STAT3 proteins were detected in the mitochondria fraction of WPMY and MEF cells using western blot analysis (Fig. 4c, e). Exposure to CdCl_2_ rapidly decreased mitoSTAT3 abundance within 30 min in MEFs and WPMY-1 cells, followed by a similar recovery phase (Supplementary Fig. 3b, c) as reported previously^40^. However, mitoSTAT3 levels continuously decreased in both WPMY-1 cells (Supplementary Fig. 3d) and MEFs (Supplementary Fig. 3e) without a recovery phase after prolonged exposure to CdCl_2_ for 24 h. In addition, exposure to increasing CdCl_2_ concentrations (0–40 μM) for 24 h dose-dependently decreased mitoSTAT3 levels in WPMY-1 cells (Fig. 4c, d) and MEFs (Fig. 4e, f). The levels of two mitochondrial ETC components, ATP5A and NDUFA9, were not altered in the mitochondrial fraction, suggesting that mitochondrial mass was not the leading cause of decreased mitoSTAT3 levels (Fig. 4c, e). Consistent with its role in mediating STAT3’s mitochondrial translocation^34,36,40,59–61^, pSer^727^-STAT3 was detected in the mitochondrial fraction (Supplementary Fig. 3f, g). However, exposure to CdCl_2_ did not alter the levels of STAT3 in the cytoplasmic fraction (Fig. 4g, h). Notably, melatonin pre-treatment for 1 h suppressed the decrease in mitoSTAT3 levels caused by prolonged exposure to CdCl_2_ for 24 h in WPMY-1 cells (Fig. 4i, j) and MEFs (Fig. 4k, l), suggesting that melatonin attenuates the toxic effects of CdCl_2_ by increasing mitoSTAT3 levels.

We also transfected WPMY-1 and MEF STAT3-/- cells with a transgene encoding GFP-tagged STAT3 and stained using MitoView 633 dye^62^. WPMY-1 cells showed GFP signals throughout the cytoplasm. Treatment with CdCl_2_ or melatonin did not induce noticeable changes in the colocalization of GFP-STAT with mitochondria. This could be due to the high-level expression of the GFP-STAT3 transgenes (Supplementary Fig. 3h). In contrast, MEF STAT3-/- cells transfected with the GFP-STAT3 transgene showed decreased colocalization between GFP-STAT3 and mitochondria by CdCl_2_ exposure (Supplementary Fig. 3i). Pretreatment with melatonin reduced this change.

### Melatonin does not alter STAT3 phosphorylation at Ser^727^ under Cd exposure

**Fig. 5:**
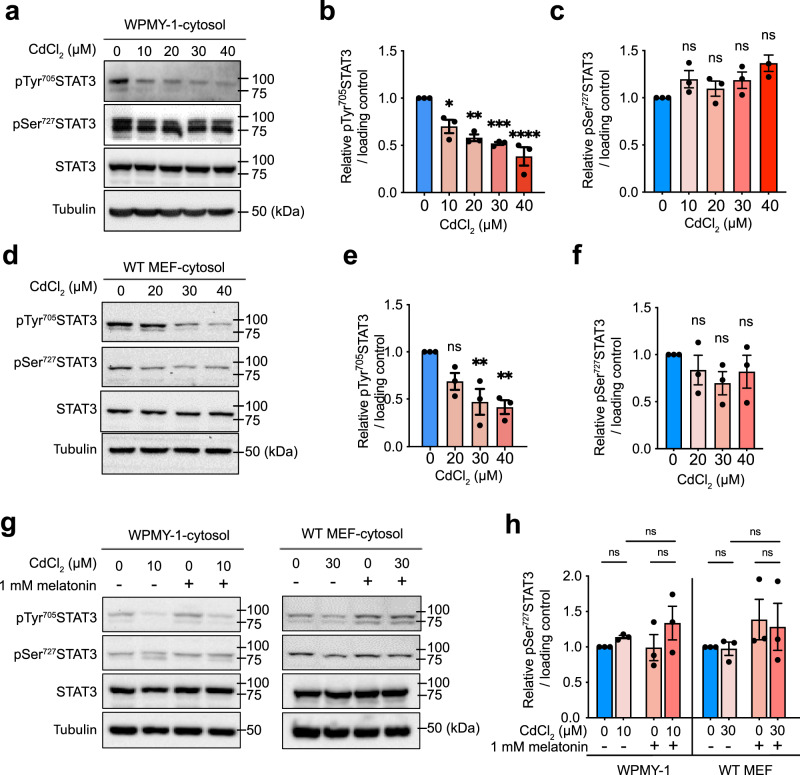
Melatonin requires new protein synthesis to restore mitoSTAT3 levels but does not affect Ser727 phosphorylation status under Cd exposure. Representative immunoblots (**a**) and quantifications of pTyr705 (**b**) and pSer727 STAT3 (**c**) in the cytosolic extracts of WPMY-1 cells stimulated with CdCl_2_. STAT3 expression was normalized to the tubulin loading control. Representative immunoblots (**d**) and quantification of pTyr705 (**e**) or pSer727 STAT3 (**f**) in the cytosolic extracts of MEFs stimulated with CdCl_2_. Representative immunoblots (**g**) and quantification of pTyr705 or pSer727 STAT3 (**h**) in the cytosolic extract of cells pretreated with 1 mM melatonin for 1 h before exposure to the indicated concentration of CdCl_2_. Data represent the mean ± SEM of at least 3–4 independent assays. *p < 0.05, ***p < 0.001; one-way ANOVA with Tukey’s post hoc test.

To elucidate the mechanism by which CdCl_2_ affects mitoSTAT3 levels, we analyzed the phosphorylation status of Tyr^705^ and Ser^727^ in cytoplasmic STAT3 from cells exposed to CdCl_2_ for 24 h. Although the rescue experiment provided no evidence for the critical role of pTyr^705^STAT3 (Fig. 4a, b), exposure to a high concentration of CdCl_2_ (40 µM) decreased pTyr^705^ levels in the cytoplasmic fraction of WPMY-1 cells (Fig. 5a, b) and MEFs (Fig. 5d, e). Despite the functional significance of pSer^727^ (Fig. 4a, b) and its apparent downregulation in the mitochondrial fraction during CdCl_2_ exposure in a concentration-dependent manner (Supplementary Fig. 3f, g), we found that cytoplasmic pSer^727^ levels were not altered in either WPMY-1 cells (Fig. 5a, c) or MEFs (Fig. 5d, f). Moreover, melatonin pretreatment for 1 h before CdCl_2_ exposure did not increase pSer^727^ levels in the cytosolic fractions of WPMY-1 cells or MEFs compared to the corresponding untreated cells (Fig. 5g, h). Together, these results suggest that Cd exposure and melatonin do not modulate mitoSTAT3 levels by enhancing phosphorylation at Ser^727^.

### Cadmium and melatonin alter the mitochondrial level of GRIM-19, a transporter of STAT3 into mitochondria

**Fig. 6:**
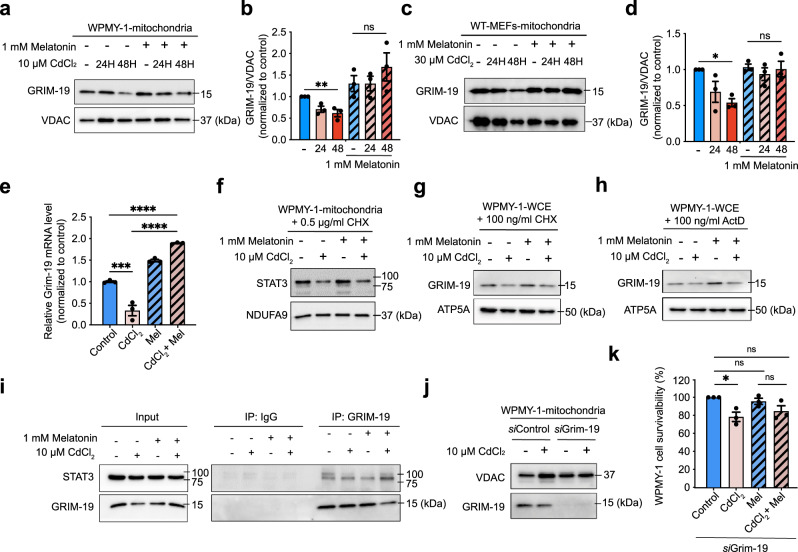
Melatonin requires new protein synthesis to prevent mitoSTAT3 loss after Cd exposure. Representative immunoblot (**a** and **c**) and quantification analysis (**b** and **d**) of GRIM-19 expression in cells incubated with melatonin and stimulated with CdCl_2_ for 24 h. **e** RT-qPCR analysis of Grim-19 mRNA levels in WPMY-1 cells. **f** Representative immunoblot of mitochondrial STAT3 expression in WPMY-1 cells incubated with cycloheximide (CHX) or melatonin and stimulated with CdCl_2_. ATP5A and Grim-19 expression in whole-cell extracts from WPMY-1 cells incubated with CHX (**g**) or actinomycin D (ActD) (**h**) and stimulated with CdCl_2_ for 24 h. Blots are representative of three independent experiments. **i** IP with GRIM-19. **j** Representing immunoblot of GRIM-19 expression in cells with *si*Grim-19. **k** Cell viability after exposure to 20 µM CdCl_2_ for 24 h in WPMY-1 cells with *si*Grim-19. *p < 0.05, **p < 0.005, ***p < 0.001,  ****p < 0.0001 ; one-way ANOVA with Tukey’s post hoc test.

Since STAT3 lacks a mitochondrial localization sequence, additional factors are required to transport STAT3 from the cytoplasm into mitochondria^63–65^. GRIM-19, a subunit of mitochondrial complex I, is a chaperone that binds to pSer^727^STAT3 to mediate its translocation from the cytoplasm to mitochondria^63,66^. We found that prolonged exposure to CdCl_2_ decreased GRIM-19 protein levels in the mitochondrial fraction of WPMY-1 cells (Fig. 6a, b and Supplementary Fig. 3d) and MEFs (Fig. 6c, d, and Supplementary Fig. 3e). Meanwhile, melatonin pretreatment for 1 h before prolonged CdCl_2_ exposure restored GRIM-19 levels in both WPMY-1 cells (Fig. 6a, b) and MEFs (Fig. 6c, d). RT-qPCR analysis further indicated that Grim-19 mRNA levels were decreased by CdCl_2_ exposure but increased by melatonin (Fig. 6e).

The altered Grim-19 mRNA levels were interesting because previous studies have indicated that the recovery of mitoSTAT3 abundance after acute and mild oxidative stress requires new protein synthesis^40^. Therefore, we monitored mitoSTAT3 levels after inhibiting de novo protein synthesis by pretreating cells with cycloheximide (CHX), a translation inhibitor, 1 h before CdCl_2_ exposure^67^. CHX suppressed the melatonin-induced enhancement of mitoSTAT3 levels (Fig. 6f and Supplementary Fig. 4a). Neither CHX nor melatonin altered STAT3 levels in the cytoplasmic fractions (Supplementary Fig. 4b, c). Interestingly, inhibiting de novo protein synthesis by pretreating cells with CHX (Fig. 6g and Supplementary Fig. 4d) and actinomycin D (ActD) (Fig. 6h and Supplementary Fig. 4e) also suppressed the recovery of Grim19 levels by melatonin. These results suggest that melatonin requires new protein synthesis to preserve mitoSTAT3 and Grim19 amounts against cadmium treatment.

Next, we investigated whether melatonin could affect the intracellular interaction between endogenous STAT3 and GRIM-19, as GRIM19 is known to bind and import STAT3 proteins into the mitochondria^63,66^. Under basal conditions, STAT3 co-precipitated with GRIM-19, suggesting binding. However, this STAT3/GRIM-19 binding was not markedly altered by treatment with either CdCl_2_ or melatonin (Fig. 6i). Interestingly, melatonin treatment did increase STAT3/GRIM-19 binding under CdCl_2_ exposure, suggesting that melatonin could require stress to promote STAT3/GRIM-19 binding (Fig. 6i). Finally, we assess the functional significance of GRIM-19 during the beneficial effects of melatonin on cell viability following CdCl_2_ exposure by using an RNAi strategy to inhibit GRIM-19 expression (Fig. 6j). Notably, *si*Grim-19 knockdown did not significantly suppress the improvement in cell survival induced by melatonin (Fig. 6k). These results show that melatonin can increase GRIM-19 expression and the mitoSTAT3/GRIM-19 interaction following Cd exposure. However, GRIM-19 is not essential to mediate the protective effects of melatonin against CdCl_2_.

### Melatonin decreases the oxidative stress-induced mitoSTAT3 and CypD interaction after Cd exposure

**Fig. 7:**
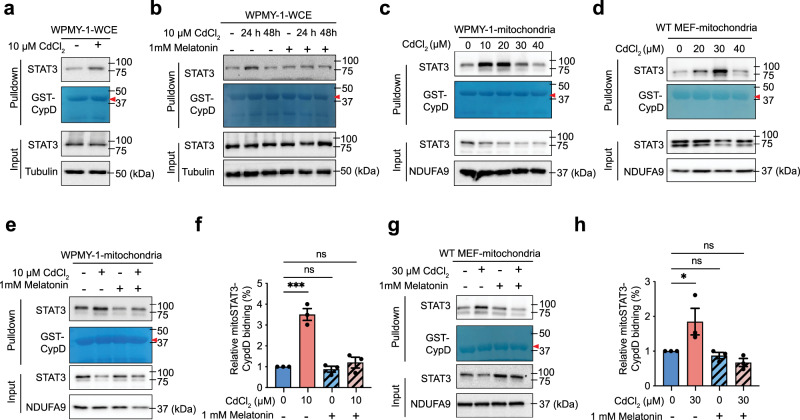
Melatonin suppresses the interaction between mitoSTAT3 and CypD after Cd treatment. **a** STAT3/CypD interaction in the whole-cell extracts of WPMY-1 cells treated with 10 µM CdCl_2_. **b** STAT3/CypD interaction in the whole-cell extracts of WPMY-1 cells treated with melatonin for 1 h before Cd exposure for the indicated time. Changes in STAT3 abundance in mitochondrial lysates from WPMY-1 cells (**c**) or WT MEFs (**d**) treated with increasing CdCl_2_ concentrations. STAT3/CypD interaction in mitochondrial lysates from WPMY-1 cells (**e**) and MEFs (**g**) treated with melatonin for 1 h before CdCl_2_ exposure. **f**, **h** Quantification of results in (**e**) and (**g**). Binding of CypD to mitoSTAT3 was quantitated and normalized to the untreated control group. Data represent the mean ± SEM of at least 3–4 independent assays. *p < 0.05, ***p < 0.001; one-way ANOVA with Tukey’s post hoc test. Blots are representative of three independent experiments.

Oxidative stress enhances STAT3 binding with Cyclophilin D (CypD), which resides in the mitochondrial matrix and acts as an mPTP activator^40,68^. This oxidative stress induced-STAT3/CypD binding in the mitochondria is suggested to be important for stabilizing the mitoSTAT3 pool and inhibiting CypD-mediated mPTP opening^40,57,69^. Therefore, we investigated whether melatonin affected mitoSTAT3 stability by regulating STAT3/CypD binding in the mitochondria. STAT3/CypD binding substantially increased following exposure to low concentrations of CdCl_2_ (10 μM) for 24 h (Fig. 7a), but not when exposed to a low CdCl_2_ concentration for a prolonged period (48 h; Fig. 7b) or a high CdCl_2_ concentration (40 μM; Fig. 7c, d). Thus, the mitoSTAT3/CypD interaction appears to be dynamic and depends on the strength of Cd exposure. Pretreatment with melatonin suppressed the increase in mitoSTAT3/CypD binding induced by CdCl_2_ exposure (24 h) in both WPMY-1 cells (Fig. 7e, f) and MEFs (Fig. 7g, h), but not during prolonged CdCl_2_-exposure for 48 h (Fig. 7b). These results suggest that the protective effects of melatonin on the mitoSTAT3 pool and CdCl_2_-induced cytotoxicity are not mediated by increased mitoSTAT3/CypD binding.

### Melatonin prevents Cd-induced mitoSTAT3 loss in the mouse prostate

**Fig. 8:**
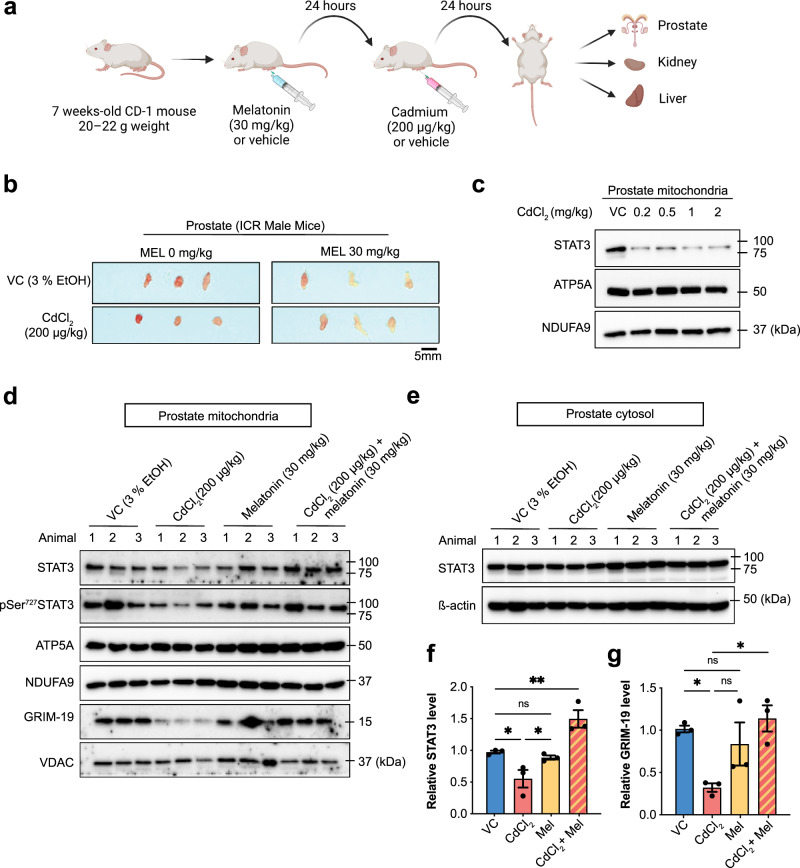
Effect of melatonin and CdCl_2_ treatment on mitoSTAT3 expression in the prostate. **a** Experimental scheme. **b** Representative images of prostate size in seven-week-old ICR male mice injected into the prostate with either the vehicle or CdCl_2_ (200 µg/kg) in the presence or absence of melatonin (30 mg/kg). **c** Representative image of STAT3 expression in the prostate after treatment with the indicated CdCl_2_ concentration. **d**–**g** Protein expression in the prostate after treatment with CdCl_2_ (200 μg/kg) with or without melatonin (30 mg/kg). Mitochondrial (**d**) or cytosolic fractions (**e**) were immunoblotted for STAT3, pS727, and Grim-19. Quantification of mitoSTAT3 (**f**) and Grim-19 (**g**) in mitochondrial fractions normalized to VDAC. Three mice were used per treatment group. Data represent the mean ± SEM of at least 3–4 independent assays. *p < 0.05, **p < 0.005; one-way ANOVA with Tukey’s post hoc test.

So far, we showed that melatonin can attenuate Cd-induced cytotoxicity in human prostate stromal cells (WPMY-1) and mouse embryonic fibroblasts (MEFs) by enhancing mitoSTAT3 function in vitro. To evaluate the effect of melatonin on CdCl_2_ toxicity in vivo, seven-week-old CD-1 ICR mice were treated with CdCl_2_ (200 µg/kg) for 24 h, followed by monitoring of prostate, liver, and kidney tissues that have been associated with Cd toxicity (Fig. 8a)^14,15,17–19^. We found that CdCl_2_ exposure notably reduced prostate tissue size compared to untreated controls (Fig. 8b). However, pre-injection with 30 mg/kg melatonin 24 h before CdCl_2_ treatment suppressed the decrease in prostate tissue size after CdCl_2_ exposure (Fig. 8a, b). No remarkable changes in tissue size were observed in the liver (Supplementary Fig. 5a) and kidney (Supplementary Fig. 5b) of mice treated with melatonin and CdCl_2_. Interestingly, mitoSTAT3 levels were significantly decreased in mouse prostate tissues treated with increasing CdCl_2_ concentrations (Fig. 8c). Melatonin inhibited the reduction in total mitoSTAT3 and pSer727-mitoSTAT3 levels after CaCl_2_ treatment (Fig. 8d, f) while also suppressing the reduction in mitochondrial GRIM-19 levels following CdCl_2_ exposure (Fig. 8d, g). However, neither CdCl_2_ nor melatonin altered cytoplasmic STAT3 levels (Fig. 8e). CdCl_2_ did not affect the mitochondrial proteins NDUFA9 and ATP5A, suggesting that decreased mitochondrial content is not the main cause of decreased mitoSTAT3 levels in vivo (Fig. 8d). No significant reductions in mitoSTAT3 and GRIM-19 expression levels were observed in the liver (Supplementary Fig. 5c, e) or kidneys (Supplementary Fig. 5d, f) of mice treated with melatonin and CdCl_2_. Together, these findings suggest that melatonin could mitigate the adverse effects of CdCl_2_ on the mitochondrial STAT3 function and organ size in vivo in the prostate tissue.

## Discussion

In this study, we propose that melatonin ameliorates Cd-induced mitochondrial dysfunction and cell death by regulating STAT3 function in mitochondria in two mammalian cell lines including human prostate stromal cells and mouse embryonic fibroblasts. Cd exposure reduced the abundance of GRIM-19, which transports STAT3 to mitochondria, rather than directly affecting the phosphorylation status of STAT3^63,66^. In contrast, melatonin enhanced the expression of GRIM-19 and its binding to STAT3. Notably, GRIM-19 knockdown did not inhibit the protective effects of melatonin on Cd-induced cell death, suggesting that other pathways could drive mitoSTAT3 function. Finally, we found that melatonin exerted beneficial effects against Cd-induced toxicity in vivo by maintaining STAT3 function in the mitochondria of prostate tissue but not the liver and kidney, suggesting that melatonin has a different ability to affect mitoSTAT3 function in these tissues. As such, this study establishes a functional link between Cd toxicity, the protective functions of STAT3, and melatonin.

Previous studies have suggested that the toxic effect of Cd is due to the strong induction of oxidative stress^27^, that could lead to mPTP opening and cell death^50–53,56,70^. Previous studies using animal models suggested that melatonin can attenuate Cd-induced oxidative stress^10–13^. In this study, we also showed that melatonin diminished Cd-induced mitoROS production, mPTP opening, and cell death. Like melatonin, NAC also showed a similar protective ability against Cd exposure, suggesting that reducing Cd-induced oxidative stress could be associated with the beneficial effects of melatonin. Although melatonin is a central derivative and unique indole-3-carbinol dimer that has antioxidant activity^7,10,47,71^, it cannot directly scavenge oxidative stress^72^. Instead, its action is thought to depend on the activation of other antioxidant systems. Consistently, our results support that melatonin requires STAT3 to efficiently protect against Cd exposure since the loss of STAT3 enhanced the harmful effects of Cd and abolished the protective effects of melatonin on cell survival, mitoROS, and mPTP opening.

Multiple lines of evidence support that STAT3 in the mitochondria plays a critical role in protecting against Cd toxicity. First, our rescue assays revealed that pSer^727^, but not pTyr^705^, is required for the protective effects of STAT3 against Cd. Our results also indicate that the mitochondrial fraction contains pSer^727^STAT3 which drives the translocation of STAT3 into the mitochondria^59^. Second, we observed dynamic alterations in the abundance of STAT3 and pSer^727^STAT3 in mitochondrial extracts, with mitoSTAT3 expression rapidly reducing after low dose and short-term Cd exposure, followed by recovery. However, mitoSTAT3 levels were unable to recover after continuous and high-dose exposure and mitochondrial dysfunction was increased. Third, melatonin restored the reduced mitoSTAT3 level after Cd exposure. Previous reports have indicated that mitoSTAT3 can affect ETC complex activity, mitoROS generation, mPTP opening, and cell death^34,40,57,61,73,74^. Here, we found that melatonin can restore reduced mitoSTAT3 levels after Cd exposure and may therefore mediate the observed toxic effects of Cd exposure.

Although our rescue assays failed to demonstrate that nuclear STAT3 is required to protect against Cd exposure, we observed that Cd exposure decreased pTyr^705^ STAT3 levels in the cytosolic fraction in a dose-dependent manner and that this effect was suppressed by melatonin. Further studies are required to elucidate the role of nuclear STAT3 in the protective effects of melatonin against Cd exposure.

We found no evidence that melatonin modifies pSer^727^ status to alter the accumulation of mitoSTAT3. Interestingly, our CHX assays revealed that melatonin requires new protein synthesis to sustain mitoSTAT3 levels. CHX also affected GRIM-19, which is a chaperone protein that delivers STAT3 to the mitochondria^63,66^. GRIM-19 levels were decreased following Cd exposure and recovered by melatonin in a manner that depends on new protein synthesis. Our qPCR analysis also corroborated the regulation of GRIM-19 expression by melatonin. As previously revealed, we also found that GRIM-19 binds to STAT3 in human prostate stromal cells and mouse embryonic fibroblasts^63,66^. Notably, this binding was decreased by Cd exposure and increased by melatonin treatment. However, we could not directly determine the functional relevance of GRIM-19 in the ability of melatonin to reduce cytotoxicity upon Cd exposure because RNAi against GRIM-19 did not significantly suppress the protective effects of melatonin against Cd exposure. Therefore, there may be other mechanisms that mediate the entry of STAT3 into mitochondria in the absence of GRIM-19.

In mouse prostate tissue, we found that Cd exposure reduced prostate size and that melatonin pretreatment suppressed this effect. In addition, mitoSTAT3 expression was reduced by Cd exposure and recovered by melatonin, suggesting that the mechanisms through which melatonin protects against Cd may be conserved in cultured prostate stromal cells and in vivo. GRIM-19 levels were also altered by Cd and melatonin in vivo, as observed in cultured cells. Interestingly, our results indicated that the liver and kidney of these animals have no changes in their size and mitoSTAT3 and GRIM-19 levels in response to Cd and melatonin. Thus, these three tissues have a different in vivo ability to modulate mitoSTAT3 function in response to Cd exposure. However, our in vitro assays focus on non-liver and kidney cells. Thus, further studies are required to evaluate the general role of STAT3 and melatonin in these tissues against Cd exposure.

In summary, our data demonstrate that melatonin suppresses Cd cytotoxicity by enhancing the mitochondrial localization of STAT3 to improve mitochondrial homeostasis and cell survival. Moreover, we propose a new role for melatonin in the regulation of GRIM-19 levels, which has been known to play a critical role in mitoSTAT3 translocation and mitochondrial homeostasis^63,66^. STAT3 (-/-) mice have a shortened lifespan, dysfunctional/dysregulated mitochondrial function, and excessive ROS^73^, treatment with a ROS scavenger extended the lifespan suggesting that STAT3 plays crucial role in ROS homeostasis^73^. Since melatonin is generally accepted as safe for short-term use without causing dependence^75^, our results suggest that melatonin could be a potential strategy to regulate mitochondrial STAT3 function to attenuate Cd-associated pathological conditions.

## Methods

### Cell culture

The following cell lines were used in this study: wild-type mouse embryonic fibroblasts (MEFs), STAT3^−/−^ MEFs, and WPMY-1 human prostate stromal cells. WT and STAT3^−/−^ MEFs were provided by Dr. Andrew Larner (Virginia Commonwealth University, Virginia, U.S.A). WPMY-1 cells were purchased from the ATCC (#CRL-2854). Cells were grown in an appropriate growth culture medium (Dulbecco’s Modified Eagle’s Medium (DMEM) without sodium pyruvate) supplemented with 10% heat-inactivated fetal bovine serum (FBS; Thermo Fisher Scientific, #10082147) and penicillin-streptomycin (10,000 U/mL; Thermo Fisher Scientific, #15140122). Melatonin was pre-incubated with cells 1 h before CdCl_2_ treatment. All other inhibitors were pre-incubated with melatonin for > 2 h prior to CdCl_2_ treatment. Without melatonin, cells were pre-incubated for 1 h before CdCl_2_ treatment. Control cells were treated with DMSO and ethanol.

### Reagents

The following chemicals and inhibitors were used in this study: CdCl_2_ (Sigma, #202908), melatonin (Sigma, #M5250), CHX (protein synthesis inhibitor; 50 μg/mL, MP Biomedicals, #SKU02194527-CF), complete protease inhibitor cocktail (according to the manufacturer’s instructions; Roche, #4693159001), PhosSTOP phosphatase inhibitor cocktail (according to the manufacturer’s instructions; Roche, #4906837001), N-Acetyl Cystein (NAC, Sigma, #A9165).

### siRNAs and transfection

For the STAT3 siRNA experiments, WPMY-1 cells (50% confluence) were transfected with STAT3-specific or scramble siRNAs (20 µM) using Lipofectamine cell reagent (Thermo Fisher Scientific, #11668030), according to the manufacturer’s protocols. STAT3 siRNAs (sense - CCG AGC CAA UUG UGA UGC U(dTdT), antisense—AGC AUC ACA AUU GGC UCG G(dTdT)) were purchased from Bioneer. Grim-19 siRNA constructs were purchased from Santa Cruz Biotechnology (cat# sc-60765)^76^.

### Recombinant plasmids

The STAT3-GFP plasmid purchased from Addgene (#110495). The pGEX-CypD plasmid was gifted by Dr. Andrew Larner^17^. pGEX-CypD and pGEX vectors were transformed into BL-21(DE3)-competent bacteria. Bacterial clones containing the plasmid of interest were grown overnight in 25 mL of LB medium and then diluted (1:15) to a 500 mL culture. Bacteria were grown for 5 h until they reached an optical density of 0.6 at 600 nm, at which point protein expression was induced with 1 mM isopropyl-β-D-thiogalactopyranoside. After the bacteria had been pelleted and lysed in a bacterial lysis buffer and then sonicated, the bacterial cell extract was incubated with glutathione Sepharose 4 B beads (GE Healthcare, #17-0756-01) for 1 h at 4 °C. Bead-bound GST proteins were pelleted, washed three times in bacterial lysis buffer, and stored at −80 °C. A portion of the purified proteins was resolved using SDS-PAGE. Gels were fixed and stained with Coomassie to quantify GST recombinant proteins against a bovine serum albumin (BSA) standard curve.

### Mitochondria isolation and pull-down assays

Cells were treated with CdCl_2_ for 24 h with or without 1 mM melatonin and then washed with 1× PBS, trypsinized using TrypLE, and collected in DMEM. After harvest, pellets were resuspended 1–2 ml of sucrose buffer (10 mM Hepes (pH 7.4), 250 mM sucrose, 1 mM EDTA, and 1x protease and phosphatase inhibitor) and incubated on ice for 10 min. Cells were homogenized by a metal douncer on ice with manual strokes until cells were broken. Cells were collected and spun down at 800 × *g* for 5 min at 4 °C. For the mitochondria pellet, the supernatant was collected and spun down at 8800 *g* for 10 min at 4 °C. The crude mitochondrial pellet was resuspended in 490 μl of sucrose buffer, and 10 μl of a stock solution of trypsin (5 mg/ml) was added to each tube. Samples were then rotated for 10 min at 4 °C after adding 500 μl of 5% BSA solution to inactivate the trypsin. Samples were rotated again for 1 min at 4 °C and then spun down at 10,000 × *g* for 10 min at 4 °C. The mitochondrial pellets were washed in 500 μl of sucrose buffer. After the wash, the mitochondrial pellets were resuspended in an appropriate volume of sucrose buffer plus 1× PBS with 2% Triton X-100 plus protease and phosphatase inhibitors and stored at −80 °C until further analysis. Recombinant bead-bound GST-CypD or GST alone was blocked in H buffer [20 mM Hepes (pH 7.7), BSA (1 mg/mL), 75 mM KCl, 0.1 mM EDTA, 2.5 mM MgCl_2_, 0.05% NP-40, and 1 mM dithiothreitol (DTT)]. Lysed mitochondrial or whole-cell extracts (WCE; 250 μg) were incubated with 20 μg of bead-bound GST-CypD or GST alone and then incubated overnight at 4 °C. The beads were then pelleted and washed in H buffer, and bound proteins were subjected to immunoblot analysis.

### SDS-PAGE and western blotting

Cell samples were lysed in 20 mM HEPES (pH 7.4), 300 mM NaCl, 10 mM KCl, 1 mM MgCl_2_, 20% glycerol, and 1% Triton X-100. Equal amounts of protein were then loaded onto Tris-glycine gels and subjected to SDS-PAGE. The gels were transferred to polyvinylidene difluoride membranes (Millipore, IPVH00010) using a semi-dry transfer apparatus, blocked for 1 h in 5% milk or 5% BSA in 1× Tris-buffered saline (TBS) + 0.1% Tween 20, and incubated overnight at 4 °C with shaking with the following antibodies: STAT3 (Cell Signaling, #9139, 100 μl, diluted 1:1,000), pTyr705 STAT3 (Cell Signaling, #9131, 100 μl, diluted 1:1,000), pSer727 STAT3 (Cell Signaling, #9134, 100 ul, diluted 1:1,000), CypD (Abcam, #ab110324, 1 mg/ml, diluted 1:1,000), ATP5A (Abcam, #ab14748, 1 mg/ml, diluted 1:1,000), NDUFA9 (Abcam, #ab14713, 1 mg/ml, diluted 1:1,000), VDAC (Abcam, #ab14734, 1 mg/ml, diluted 1:1,000), Grim-19 (Abcam, #ab110240, 1 mg/ml, diluted 1:1,000), and tubulin (#T8203; Sigma-Aldrich, 2 mg/ml, 1:5,000). After incubation with secondary antibodies (anti-mouse NA931V or anti-rabbit NA934V; diluted 1:5000) for 1 h at room temperature in 5% milk in 1× TBS + 0.1% Tween 20, bands were visualized using ECL solution (GE Healthcare, RPN2106, Thermo Fisher Scientific, cat#80196) and calibrated using a ChemiDoc Imaging System (Bio-Rad, Hercules, CA, USA). Densitometric quantification was performed using ImageJ software (NIH, Bethesda, MA, USA).

### MTT (3-(4,5-dimethylthiazol-2-yl)-2,5-diphenyltetrazolium bromide) assay

WT MEFs and WPMY-1 cells were seeded in six-well plates and exposed to CdCl_2_ for 24 h with or without melatonin pretreatment 1 h before CdCl_2_-exposure. After the medium had been removed, MTT (0.5 mg/mL) was added to each well for 15 min and then dissolved in DMSO. Color intensity was measured at 540 nm using a microplate reader (SYNERGY HTX, BioTek).

### Apoptosis analysis

Apoptotic cells were detected through Alexa Fluor 488 Annexin V/ propidium iodide (PI) staining assay^77^. The quantification was performed using flow cytometry. WPMY-1 and MEF cells were seeded in 6 well plates. After 48 h, 1× 10^5^ cells were treated with or without melatonin or NAC before CdCl_2_ treatment, and then cells were stained by annexin v-FITC/PI using an apoptosis kit according to the manufacturer’s manual (ThermoFisher Scientific, #V13242). After staining, the cells were washed and analyzed using flow cytometry (CytoFLEX, Beckman Coulter).

### Measurement of ROS production

Mitochondria-derived ROS were detected in cells stained with 5 μM MitoSOX Red (Thermo Fisher, M36008) at 37 °C for 30 min. After staining, the cells were washed and analyzed using flow cytometry (CytoFLEX, Beckman Coulter).

### Measurement of mPTP opening

mPTP opening was assessed by quenching calcein-AM fluorescence with cobalt. Briefly, WT MEFs and WPMY-1 cells were treated with CdCl_2_ in DMEM for 24 h at 37 °C, washed twice with phosphate-buffered saline (PBS), and loaded with Calcein-AM (1 μM, Molecular Probes, Life Technologies) at 37 °C in the dark. After the cells had been incubated with CoCl_2_ for 15 min with HBSS buffer, the staining solution was removed and the cells were washed, resuspended in PBS, and analyzed using a flow cytometer (CytoFLEX, Beckman Coulter).

### Measurement of mitochondrial membrane potential

MEFs and WPMY-1 cells were seeded in 96-well plates and pre-treated with 1 mM melatonin and 1 mM NAC for 1 h before CdCl_2_ treatment. After exposure to CdCl_2_ for 24 h, mitochondrial membrane potential was assessed using TMRE-Mitochondrial Membrane Potential Assay Kit (Abcam, ab113852). Cells were incubated for 30 min with 1 *μ*M TMRE at 37 °C and then washed twice with PBS/0.2% BSA. After washing, fluorescence intensity was detected using a microplate reader (SYNERGY HTX, BioTek).

### Fluorescence microscopy

WPMY-1 cells were seeded on glass coverslips. After 24 h, cells were transfected with STAT3-GFP with Fugene HD transfection kit (Promega, #E2311). After 48 h, cells were treated with CdCl_2_ with or without 1 mM melatonin pretreatment. For STAT3-GFP mitochondrial localization, live cells were treated with Mitoview633 (#70055 T, 622/648 nm) red fluorescence dye for 15 min. Live cell imaging was performed using the fluorescence microscope (Olympus CKX53, pE-300 lite).

### Animal experiments

Six-week-old male CD-1 (Institute for Cancer Research; ICR) mice weighing 20–22 g were purchased from Orient Bio Company (Seongnam, Korea). The animals were housed in a temperature- and humidity-controlled animal facility under a 12 h light/dark cycle. The animals were acclimatized for one week before the experiment and were allowed free access to a commercial rodent diet and tap water. All experiments were approved by the Institutional Agricultural Animal Care and Use Committee of KIT (IACUC No.2011-0005) and were performed in accordance with the Guide for the Care and Use of Laboratory Animals. The seven-week-old mice were injected intraperitoneally with melatonin (30 mg/kg: Sigma, St. Louis, MO, USA) or vehicle as a pretreatment. After 24 h, CdCl_2_ (200 μg/kg) or vehicle was injected. Then 24 h later, all mice were sacrificed by CO_2_ inhalation, and their prostate, liver, and kidney were excised and stored at −80 °C for western blot analysis. Melatonin was prepared in ethanol at a concentration of 50 mg/mL and diluted in saline.

### Statistics and reproducibility

All data were presented as the mean ± SEM. Parametric and non-parametric data were analyzed using one-way ANOVA with Tukey’s post hoc test or using Kruskal-Wallis analysis with Dunn’s post hoc test, respectively, using Prism v.9.00 (GraphPad Software, San Diego, CA, USA). At least three independent experiments were performed to generate the displayed data.

### Reporting summary

Further information on research design is available in the Nature Portfolio Reporting Summary linked to this article.

## Supplementary information

Supplemental Information

Description of Additional Supplementary Files

Supplementary Data 1

Reporting Summary

## Data Availability

The numerical source data for graphs are available in Supplementary Data 1. Uncropped blots and the gating strategy for FACS plots are available in Supplementary Figs. 6–9. All other data are available from the corresponding author upon reasonable request.

## Code availability

No custom codes/software were generated in this study. The following publicly available software was used for data analysis. ImageJ (https://imagej.nih.gov/ij/index.html) was used for the image analysis. Graphpad prism v9 (https://www.graphpad.com/scientific-software/prism/) was used for the analysis.
