# Supplementary material for: Melatonin protects against cadmium-induced oxidative stress via mitochondrial STAT3 signaling in human prostate stromal cells
Source: Commun Biol. 2023 Feb 8;6:157. doi: 10.1038/s42003-023-04533-7 (PMC9905543; doi:10.1038/s42003-023-04533-7)
Supplement: Supplementary file 1 — Supplemental Information [file 42003_2023_4533_MOESM1_ESM.pdf]

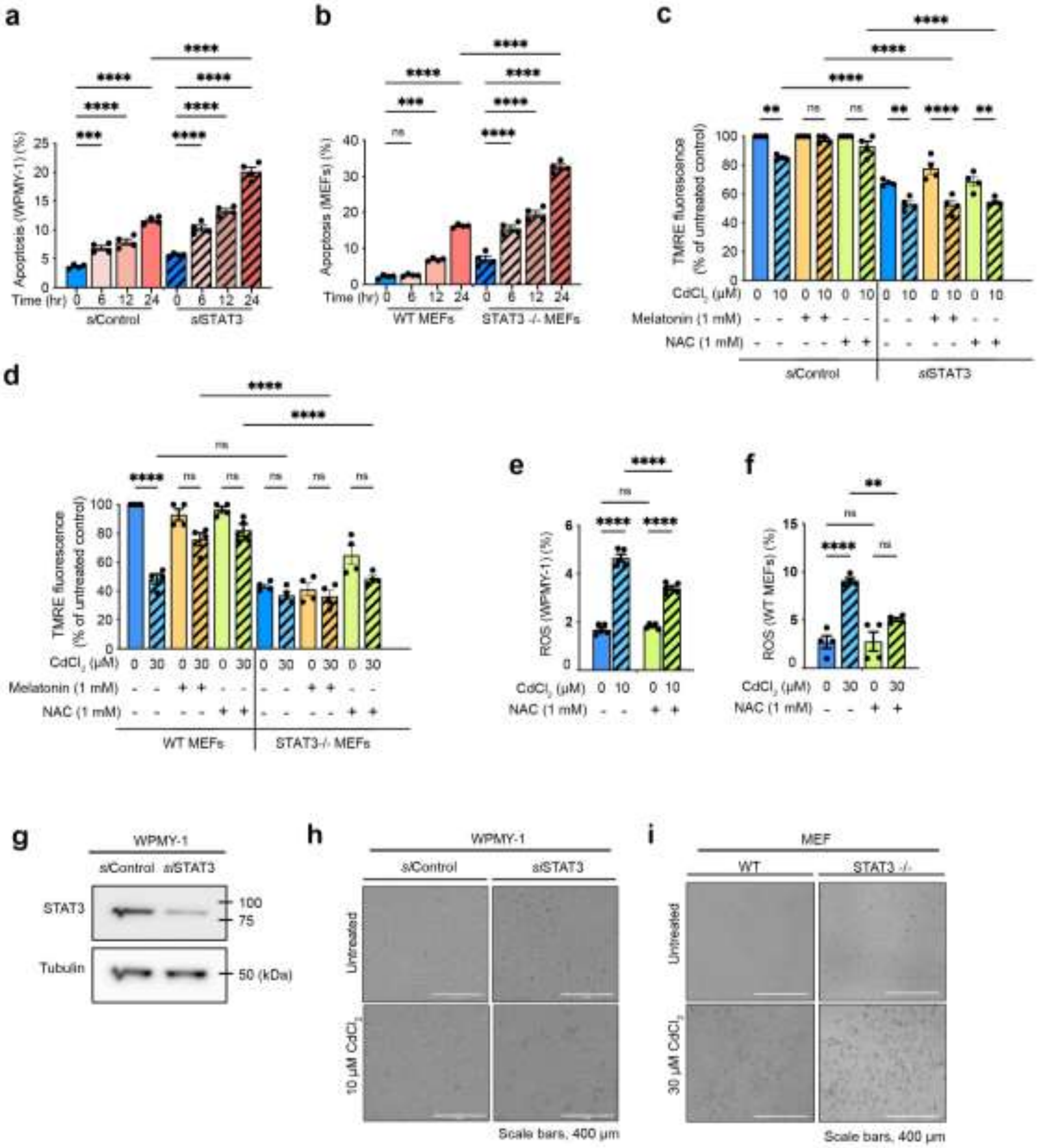

Supplementary Figure 1. Viability and mitochondria homeostasis of STAT3 siRNA-transfected WPMY-1s and STAT3$^{-/-}$ MEFs treated with $CdCl_2$. (a) Apoptotic effect of $CdCl_2$ in STAT3 siRNA transfected WPMY-1s(a) and STAT3-/- MEFs(b). The effect of $CdCl_2$ on mitochondrial potential in STAT3 siRNA transfected WPMY-1s(c) and STAT3-/- MEFs(d) with or without 1 mM melatonin or 1 mM NAC. (e) The effect of $CdCl_2$ on mitoROS in WPMY-1 cell

after 1 mM NAC pretreatment. (f) The effect of $CdCl_2$ on mitoROS in WT MEFs cell after 1 mM NAC pretreatment. (g) Representative image of STAT3 expression in WPMY-1 cells transfected with control or STAT3 siRNAs from three independent experiments. (h) Morphology of WPMY-1 cells transfected with control or STAT3 siRNAs. (i) Morphology of WT and STAT3$^{-/-}$ MEFs. Data represent the mean ± SEM of at least 3-4 independent assays. $^{**}p < 0.005$, $^{***}p < 0.001$, $^{****}p < 0.0001$; one-way ANOVA with Tukey's post hoc test.

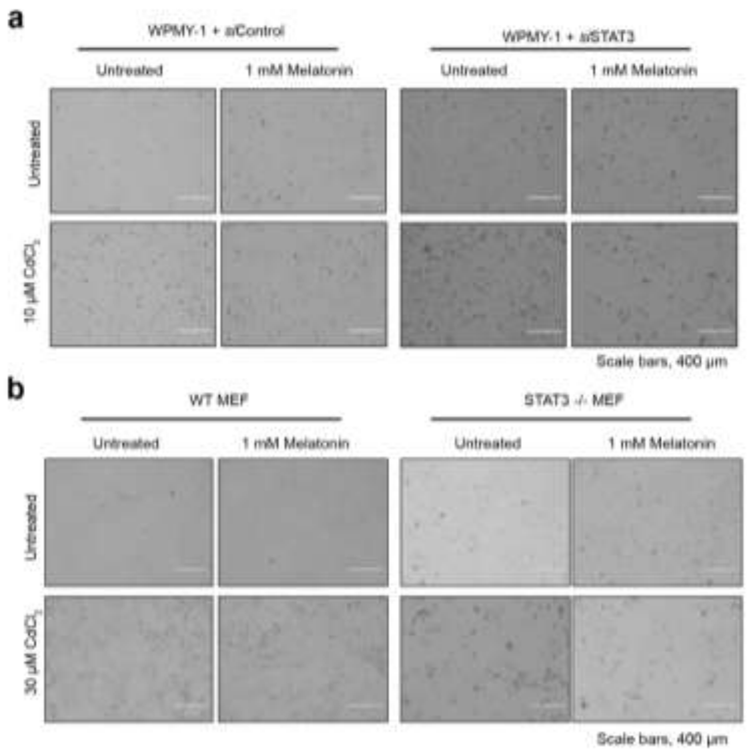

Supplementary Figure 2. Effect of melatonin on the viability of STAT3 siRNA-transfected WPMY-1s and STAT3$^{-/-}$ MEFs treated with $CdCl_2$. (a) Morphology of in WPMY-1 cells transfected with control or STAT3 siRNAs and treated with 10 μM $CdCl_2$ with or without 1 mM melatonin. (b) Morphology of WT MEFs and STAT3$^{-/-}$ MEFs treated with 30 μM $CdCl_2$ with or without 1 mM melatonin.

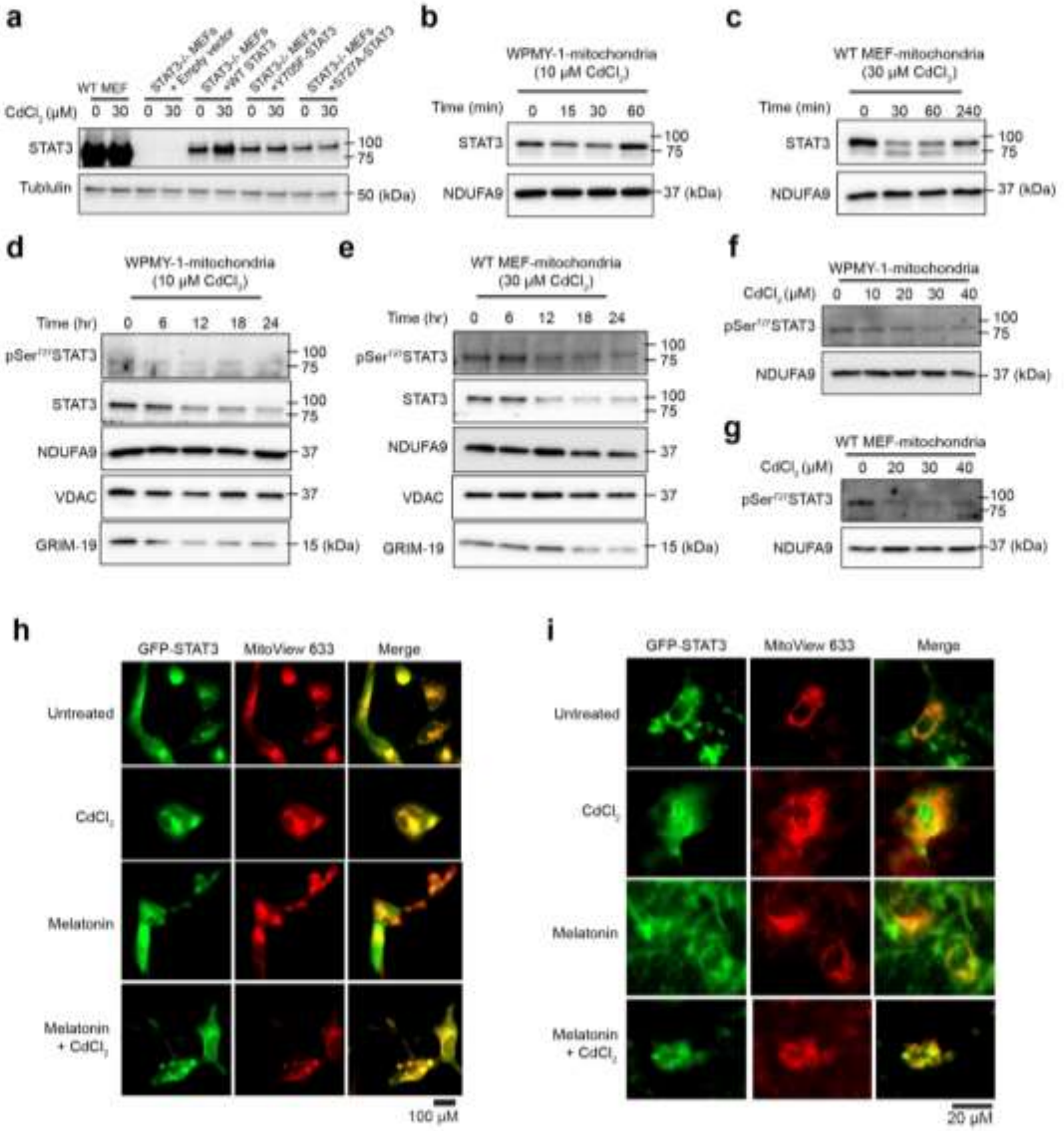

Supplementary Figure 3. Rapid loss and recovery of mitoSTAT3 after $CdCl_2$ treatment and the inhibition of STAT3 phosphorylation. (a) Immunoblots of STAT3 in WT MEFs and STAT3$^{-/-}$ MEFs, expressing various Flag-tagged STAT3 constructs and treated with $CdCl_2$. (b and c) Immunoblots of STAT3 and the loading control (NDUFA9) in mitochondrial extracts from WPMY-1 and WT MEFs treated with $CdCl_2$ for the indicated times. Immunoblots are representative of three independent experiments. (d-g) Immunoblots of pSer727-STAT3, STAT3, and NDUFA9 in mitochondrial extracts from WPMY-1s or WT MEFs treated with $CdCl_2$ for the indicated times up to 24 h (d and e) and at different concentrations (f and g). Blots are

representative of three independent experiments. (h and i) Representative images of GFP-STAT3 expression in WPMY-1 cells (h) or STAT3-/- MEFs cells (i). Mitochondria were visualized by MitoView633 red fluorescence.

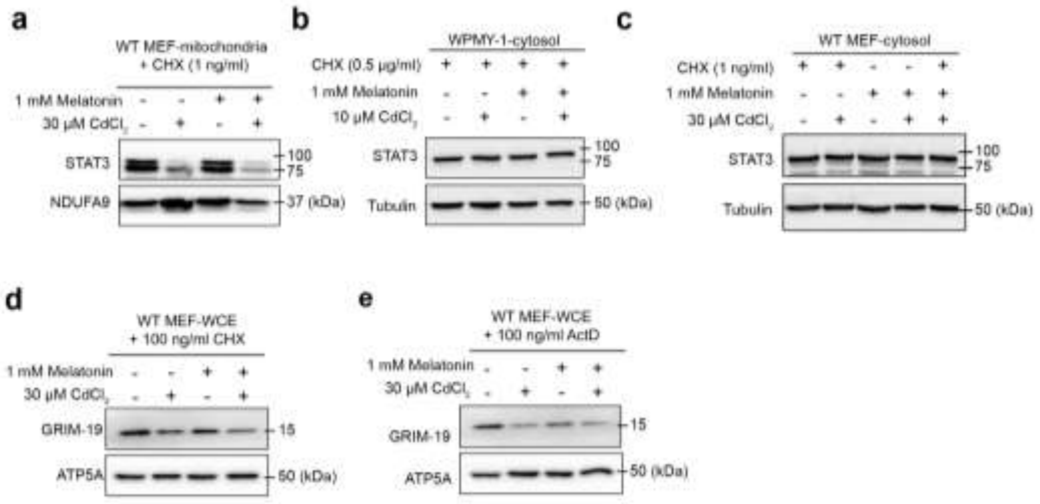

Supplementary Figure 4. Effect of cycloheximide (CHX) or actinomycin D (ActD) on STAT3 and GRIM-19. (a) Representative immunoblots of mitochondrial STAT3 expression in WT MEFs incubated with CHX or melatonin and stimulated with $CdCl_2$. (b and c) Representative immunoblots of cytosolic extracts isolated from WPMY-1 (b) and WT MEFs (c) pretreated with CHX and melatonin for 1 h prior to $CdCl_2$ stimulation. Blots are representative of three independent experiments. (d and e) ATP5A and Grim-19 expression in whole-cell extracts from WT MEFs incubated with CHX (d) or ActD (e) and stimulated with $CdCl_2$ for 24 h. Blots are representative of three independent experiments.

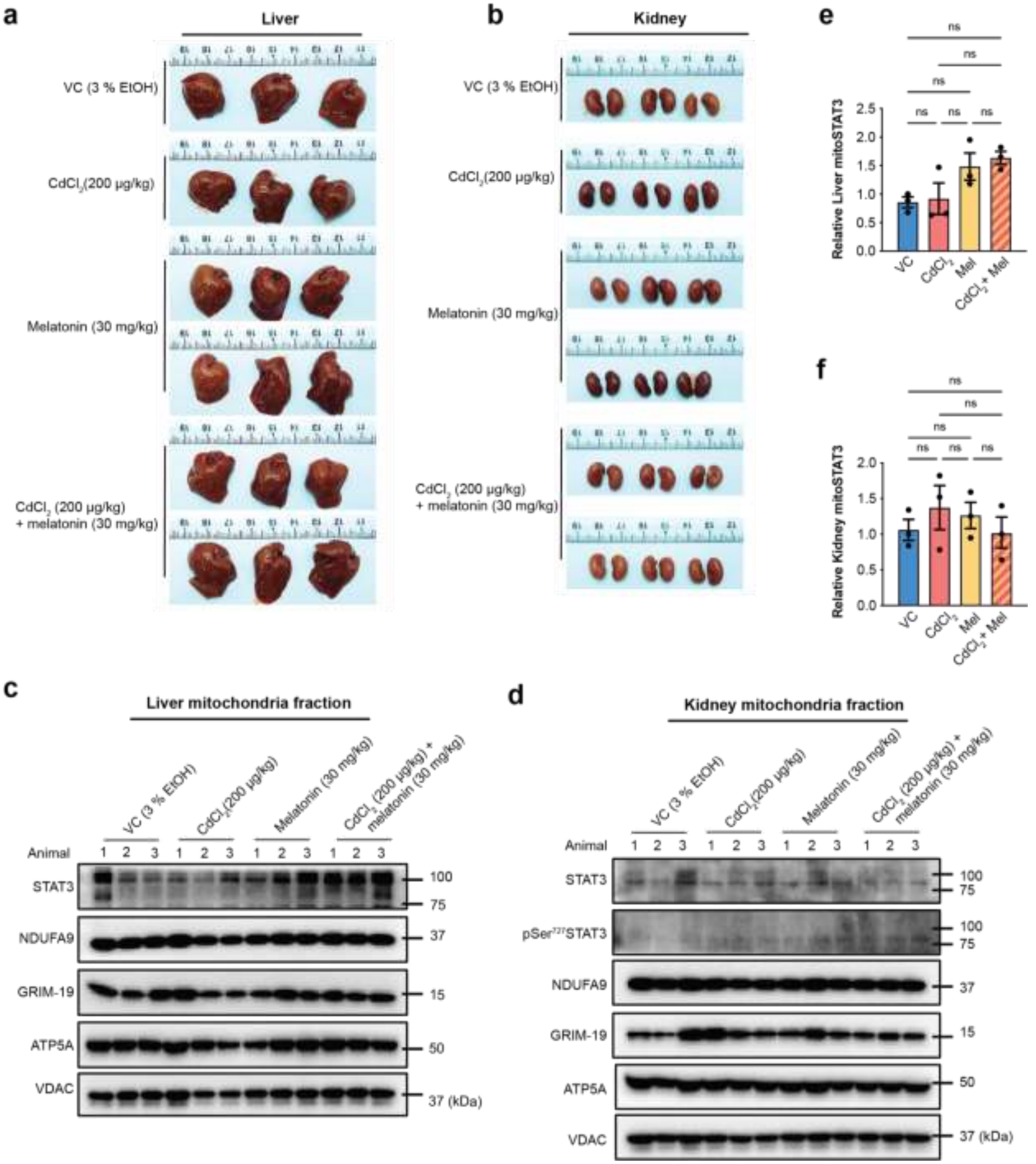

Supplementary Figure 5. *In vivo* analysis of the protective effects of melatonin against Cd-exposure. (a and b) Representative images of the liver (a) and kidney (b) size in seven-week-old ICR male mice IP injected in the prostate with the vehicle or $CdCl_2$ (200 μg/kg) in the presence or

absence of melatonin (30 mg/kg). (c and d) Mitochondrial protein expression in the liver (c) or kidney (d) of seven-week-old ICR male mice treated with $CdCl_2$ (200 μg/kg) with or without melatonin (30 mg/kg). (e and f) Quantification of the liver (e) and kidney (f) expression normalized to VDAC. Three mice were used per treatment group. Data represent the mean ± SEM. **p < 0.005, ***p < 0.001; one-way ANOVA with Tukey's post hoc test.

Related to figure 4

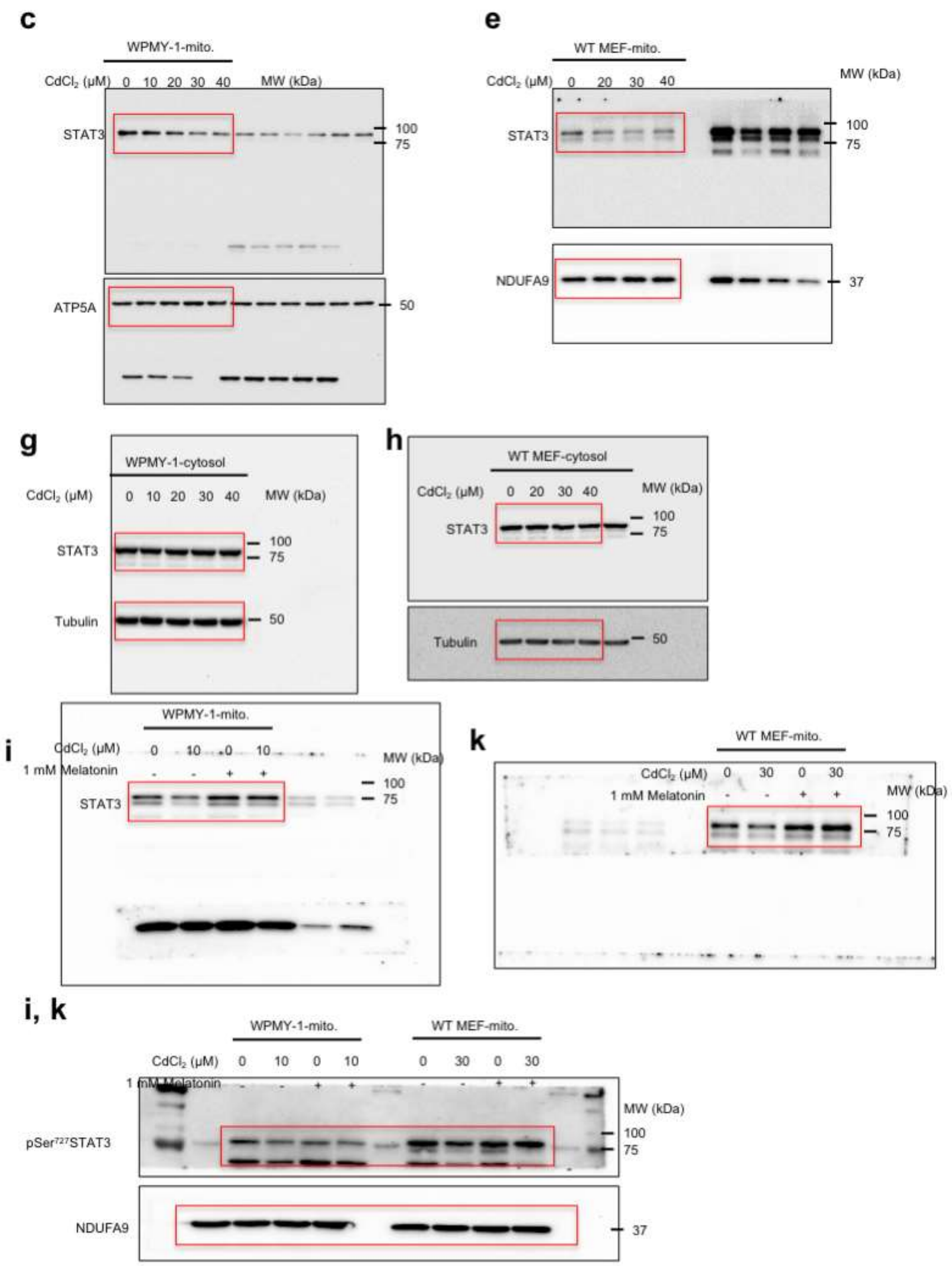

Related to figure 5

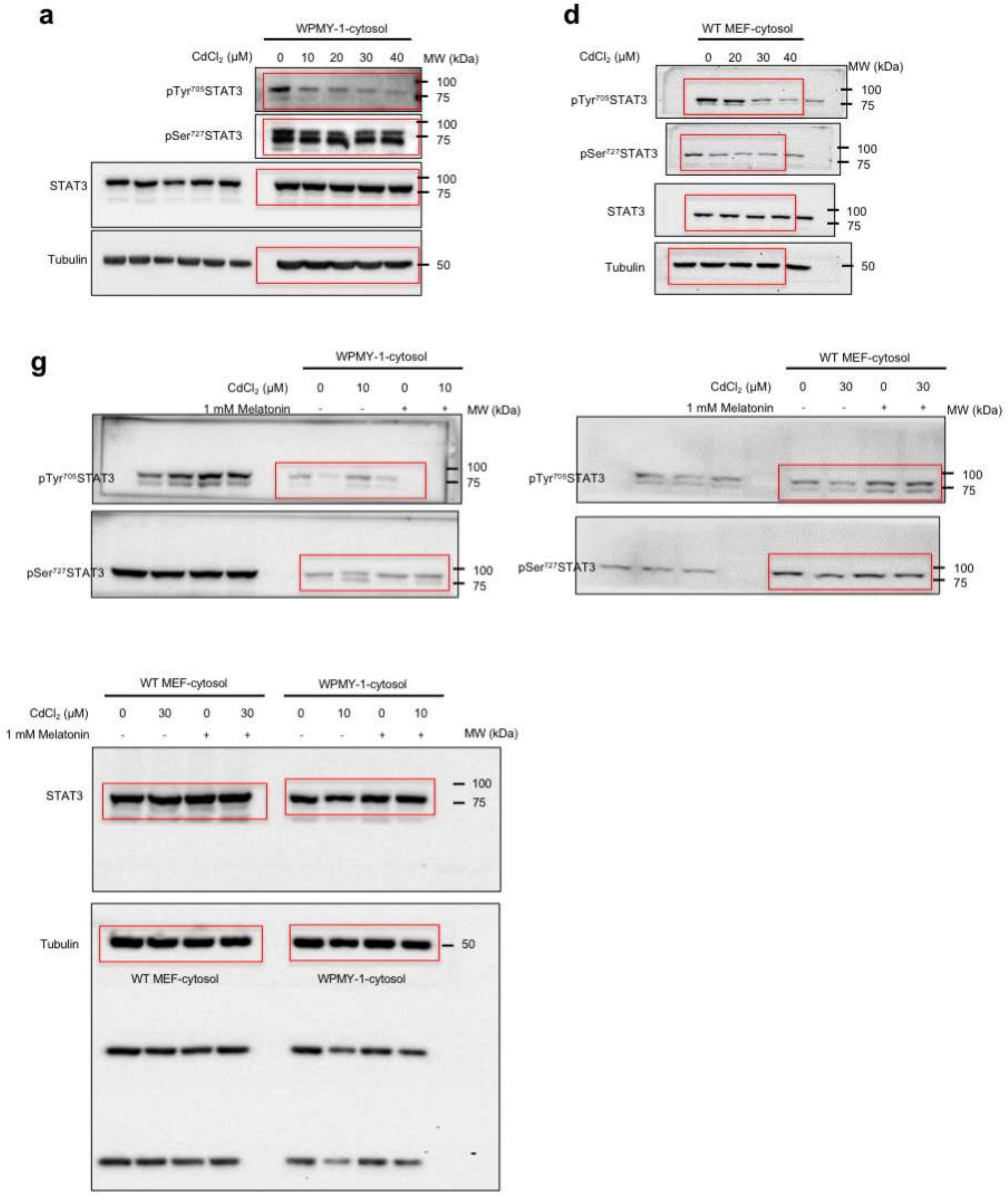

Related to figure 6

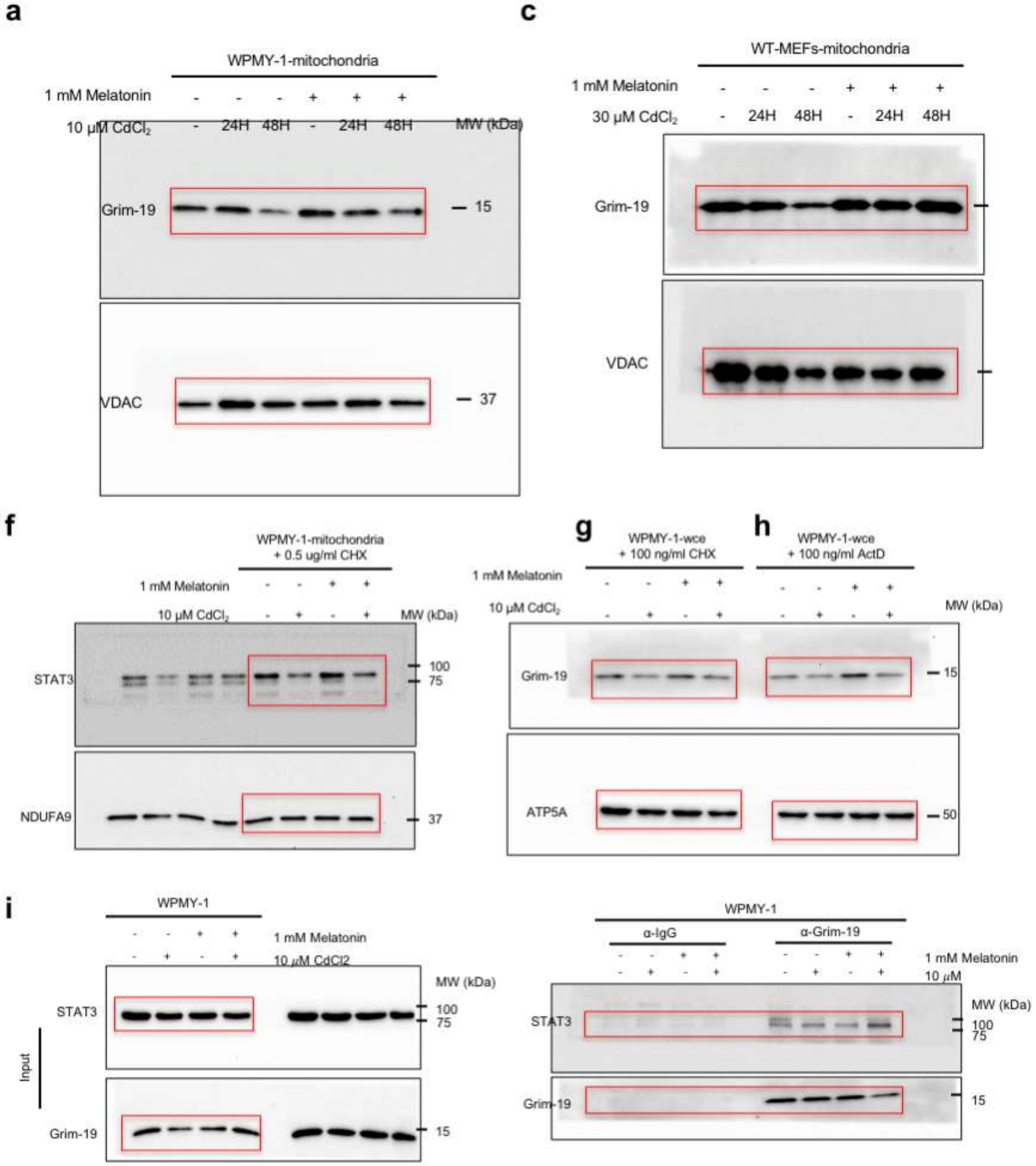

j

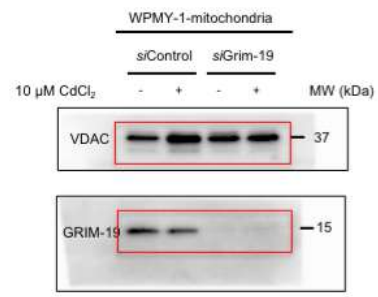
WPMY-1-mitochondria
siControl
siGrim-19
10 μM CdCl2
- + - +
MW (kDa)
VDAC
37
GRIM-19
15

Related to figure 7

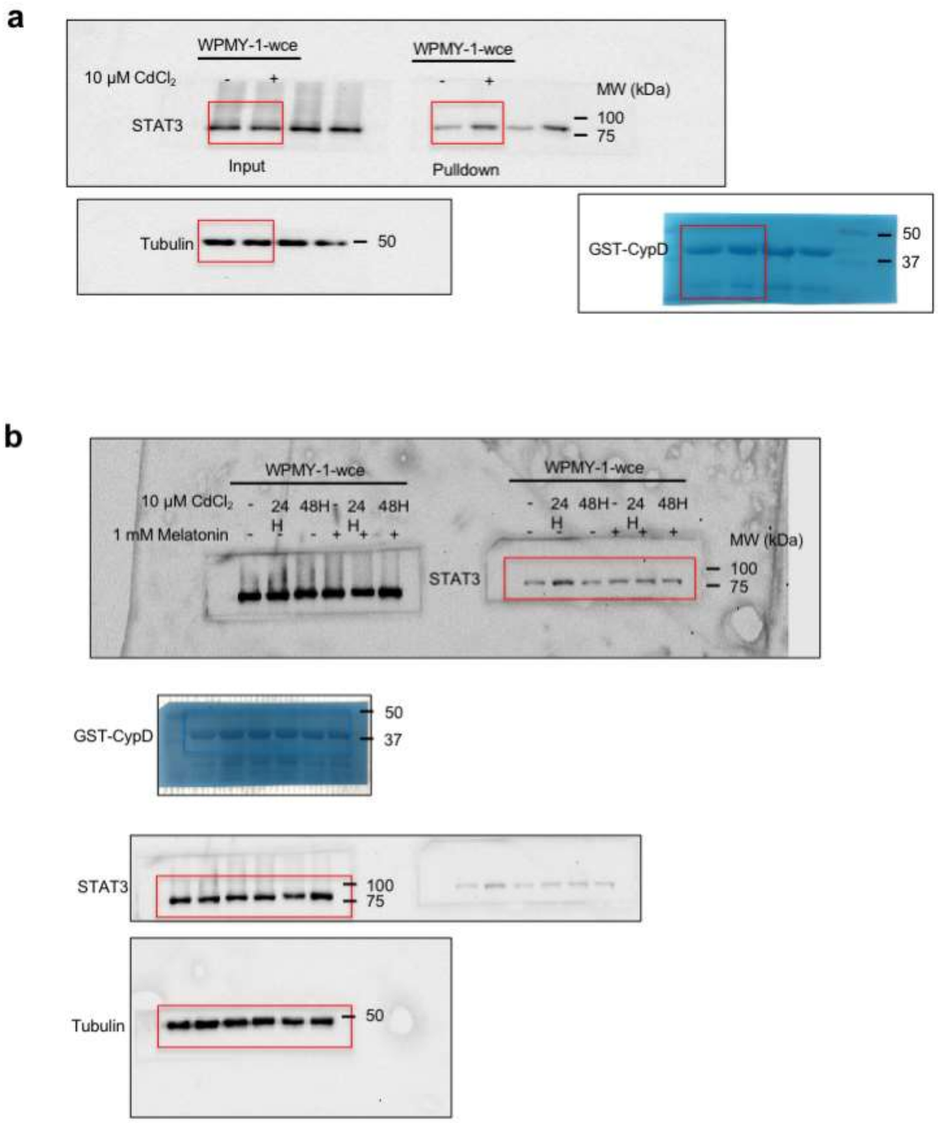

c

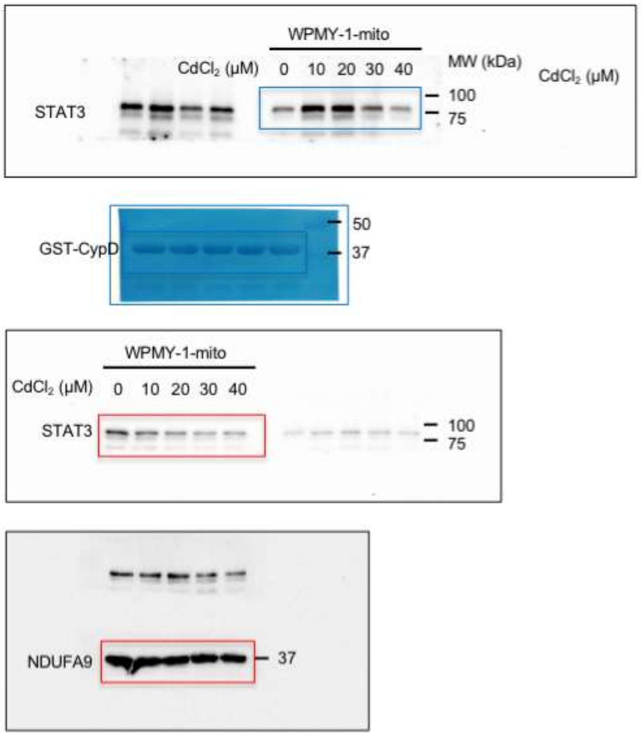

WPMY-1-mito
CdCl2 (μM)
0 10 20 30 40
MW (kDa)
STAT3
100
75
GST-CypD
50
37
NDUFA9
37

d

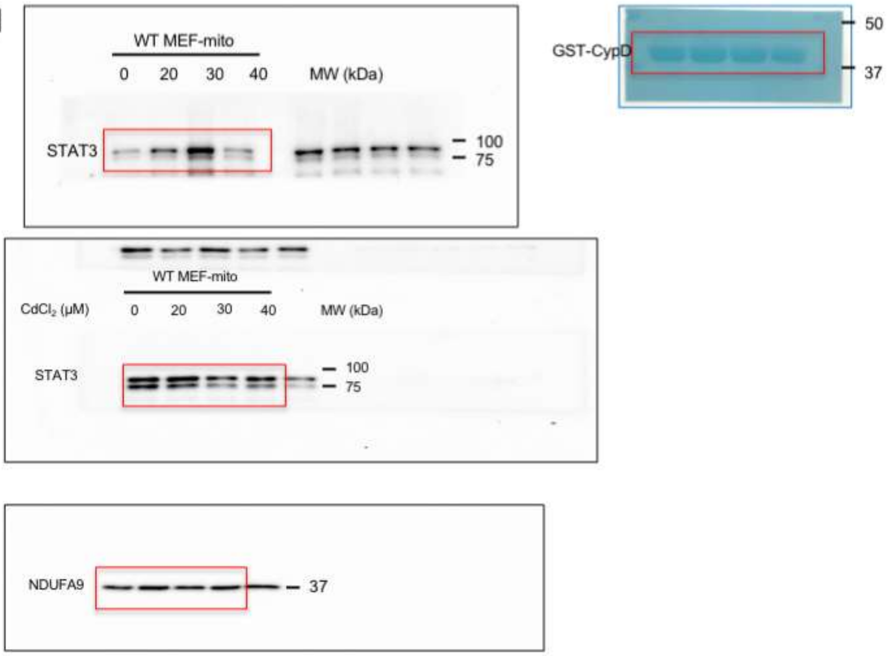

WT MEF-mito
0 20 30 40
MW (kDa)
STAT3
100
75
GST-CypD
50
37
CdCl2 (μM)
NDUFA9
37

e

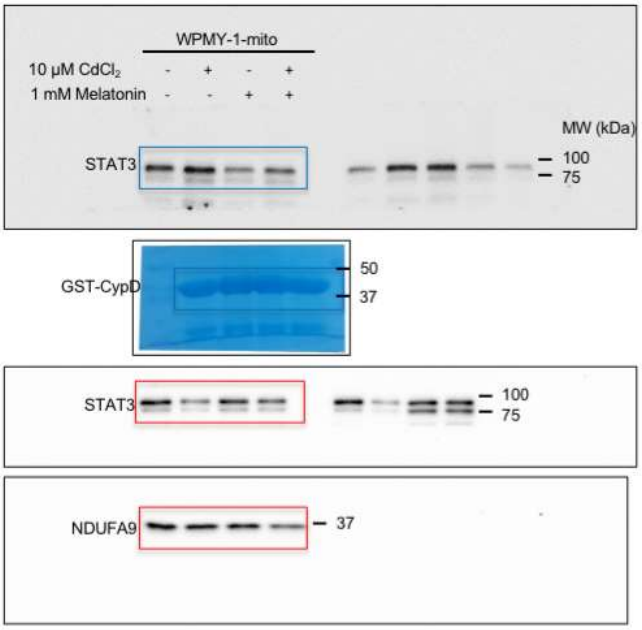
WPMY-1-mito
10 µM CdCl2 - + - +
1 mM Melatonin - - + +
MW (kDa)
STAT3
100
75
GST-CypD
50
37
STAT3
100
75
NDUFA9
37

g

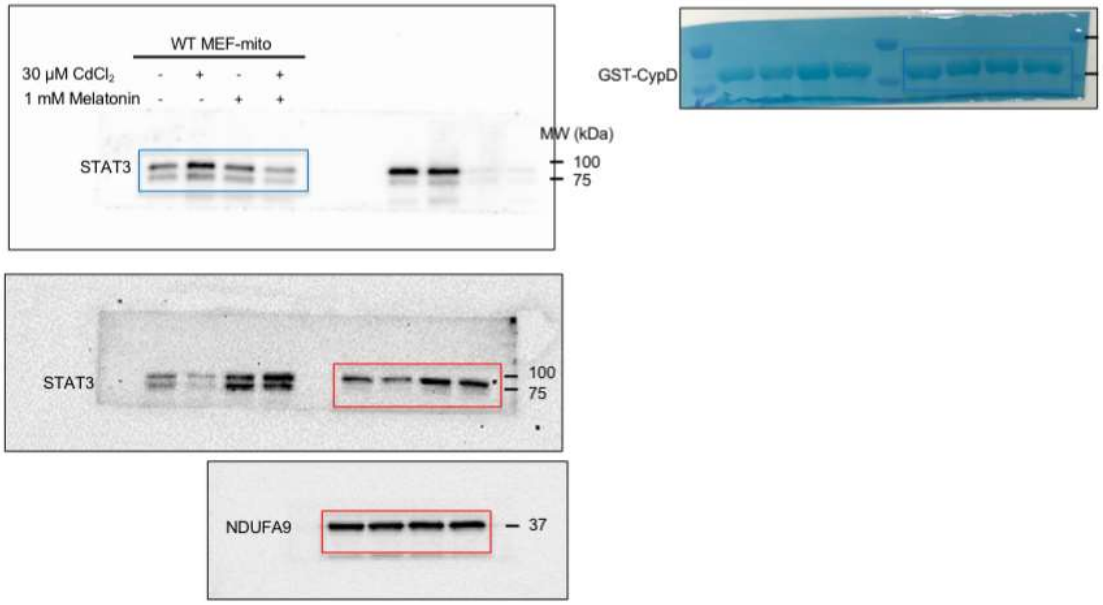
WT MEF-mito
30 µM CdCl2 - + - +
1 mM Melatonin - - + +
MW (kDa)
STAT3
100
75
GST-CypD
50
37
STAT3
100
75
NDUFA9
37

Related to figure 8

c

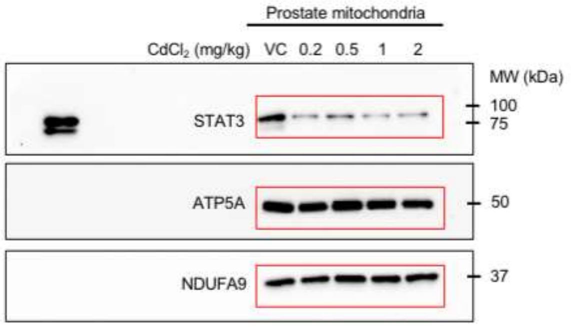

d

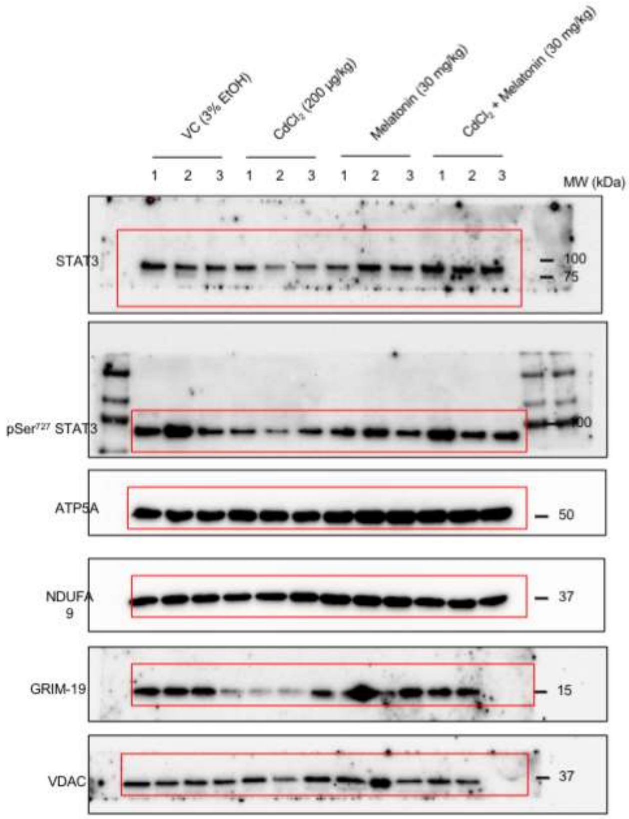

e

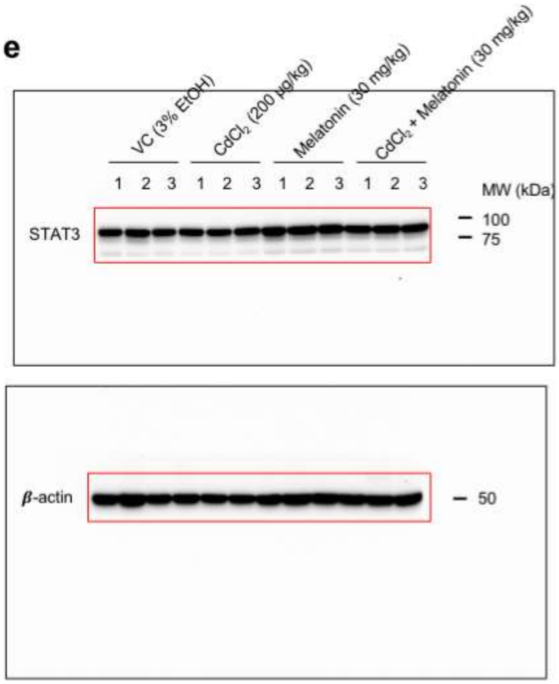

VC (3% EtOH)
CdCl2 (200 μg/kg)
Melatonin (30 mg/kg)
CdCl2 + Melatonin (30 mg/kg)
1 2 3 1 2 3 1 2 3 1 2 3
MW (kDa)
STAT3
100
75
β-actin
50

Related to supplementary data figure 1

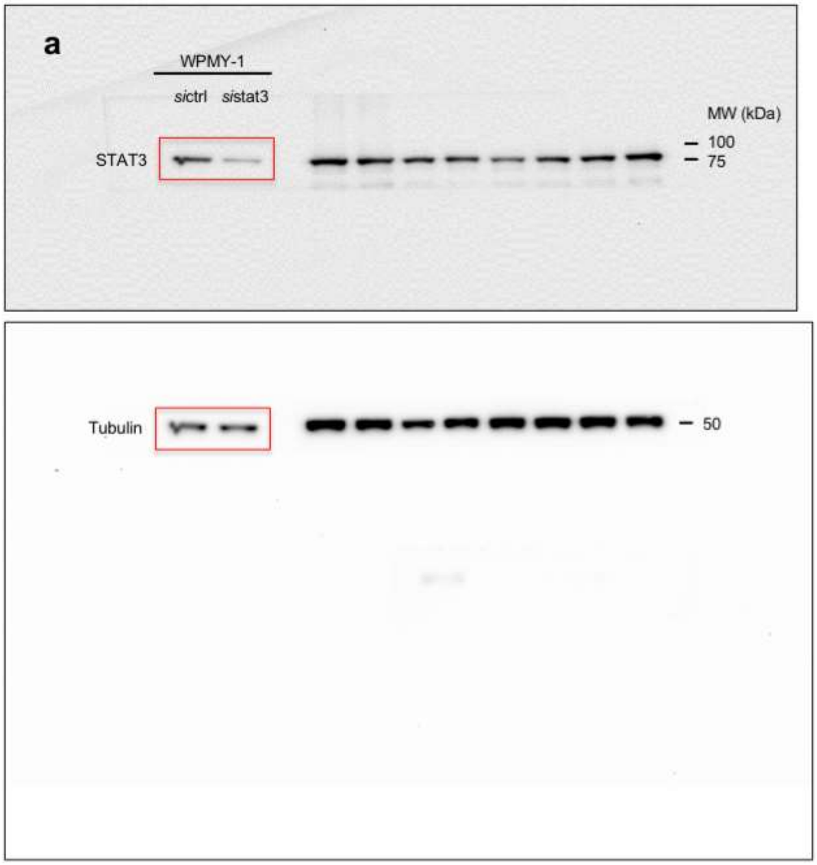

Related to supplementary data figure 3

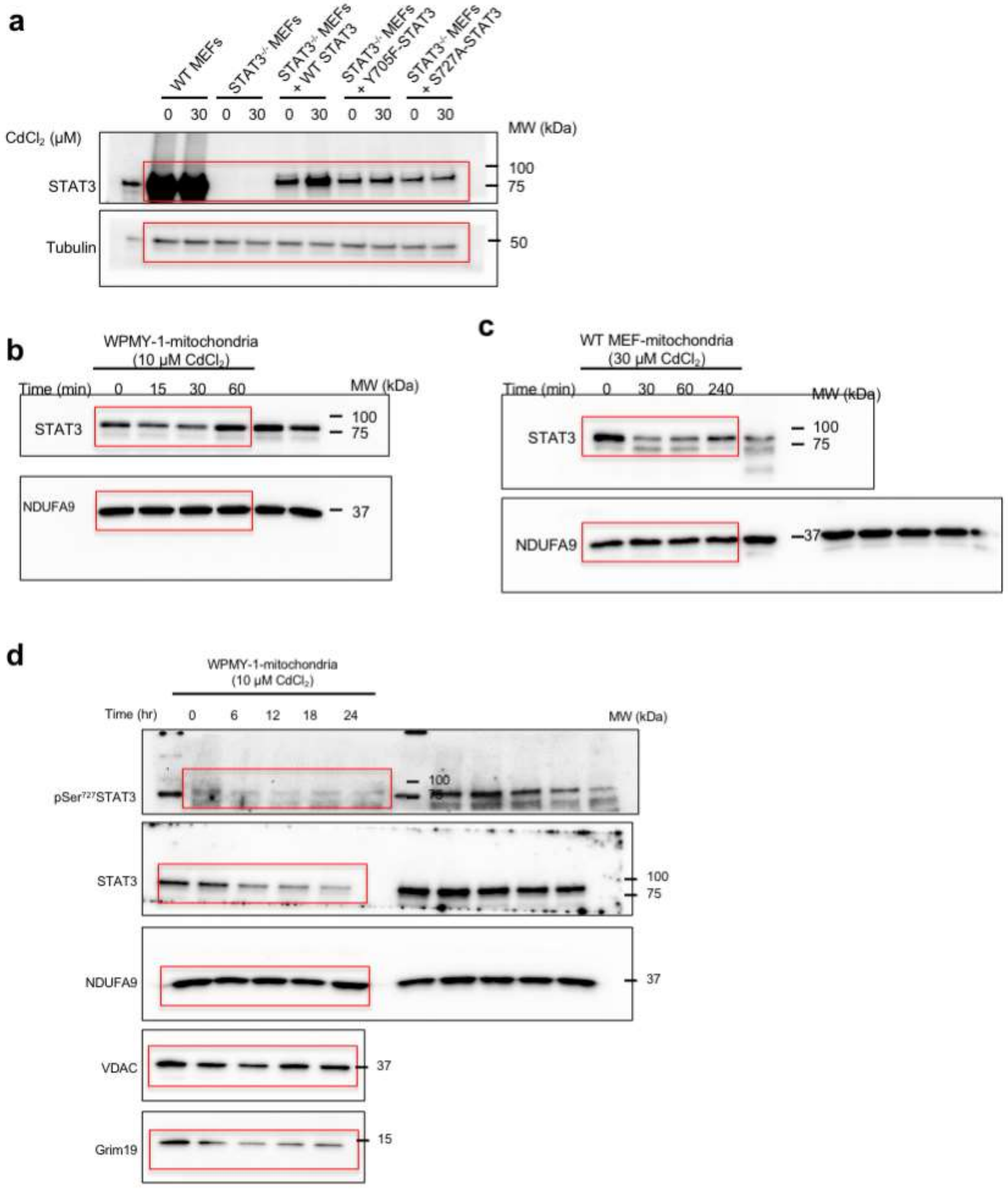

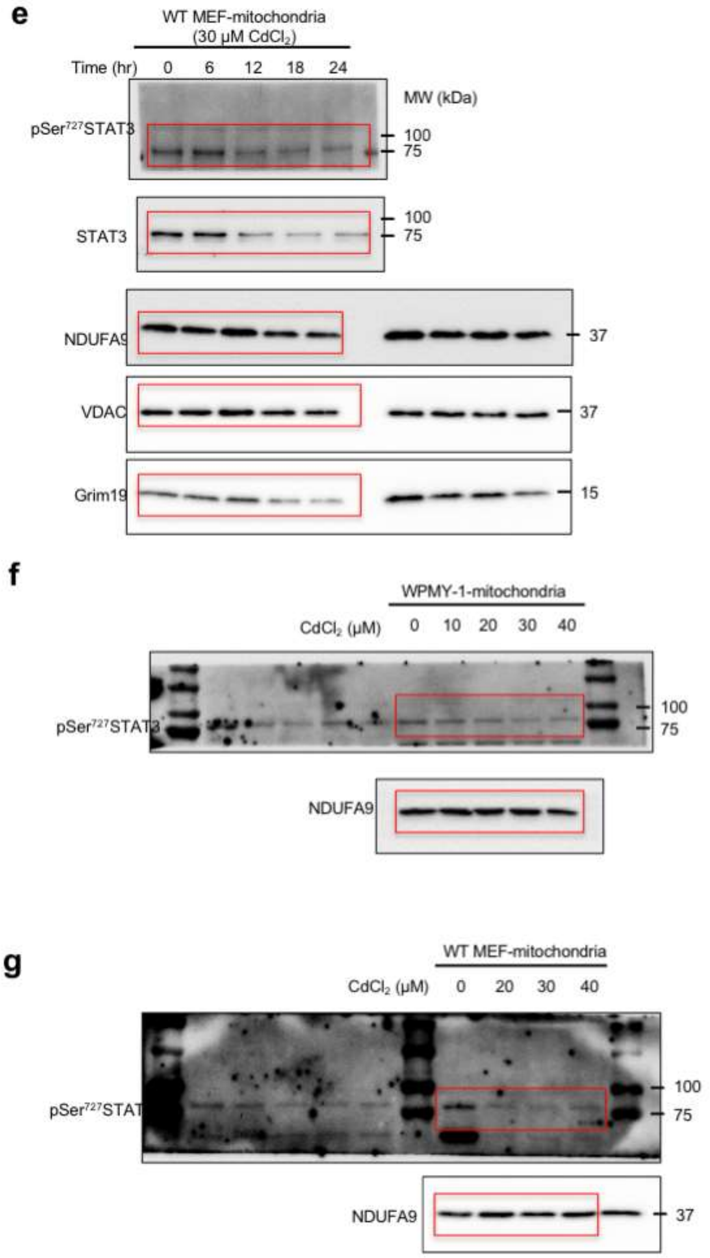
e
WT MEF-mitochondria
(30 µM CdCl2)
Time (hr) 0 6 12 18 24
MW (kDa)
pSer727STAT3
100
75
STAT3
100
75
NDUFA9
37
VDAC
37
Grim19
15
f
WPMY-1-mitochondria
CdCl2 (µM) 0 10 20 30 40
pSer727STAT3
100
75
NDUFA9
g
WT MEF-mitochondria
CdCl2 (µM) 0 20 30 40
pSer727STAT
100
75
NDUFA9
37

Related to supplementary data figure 4

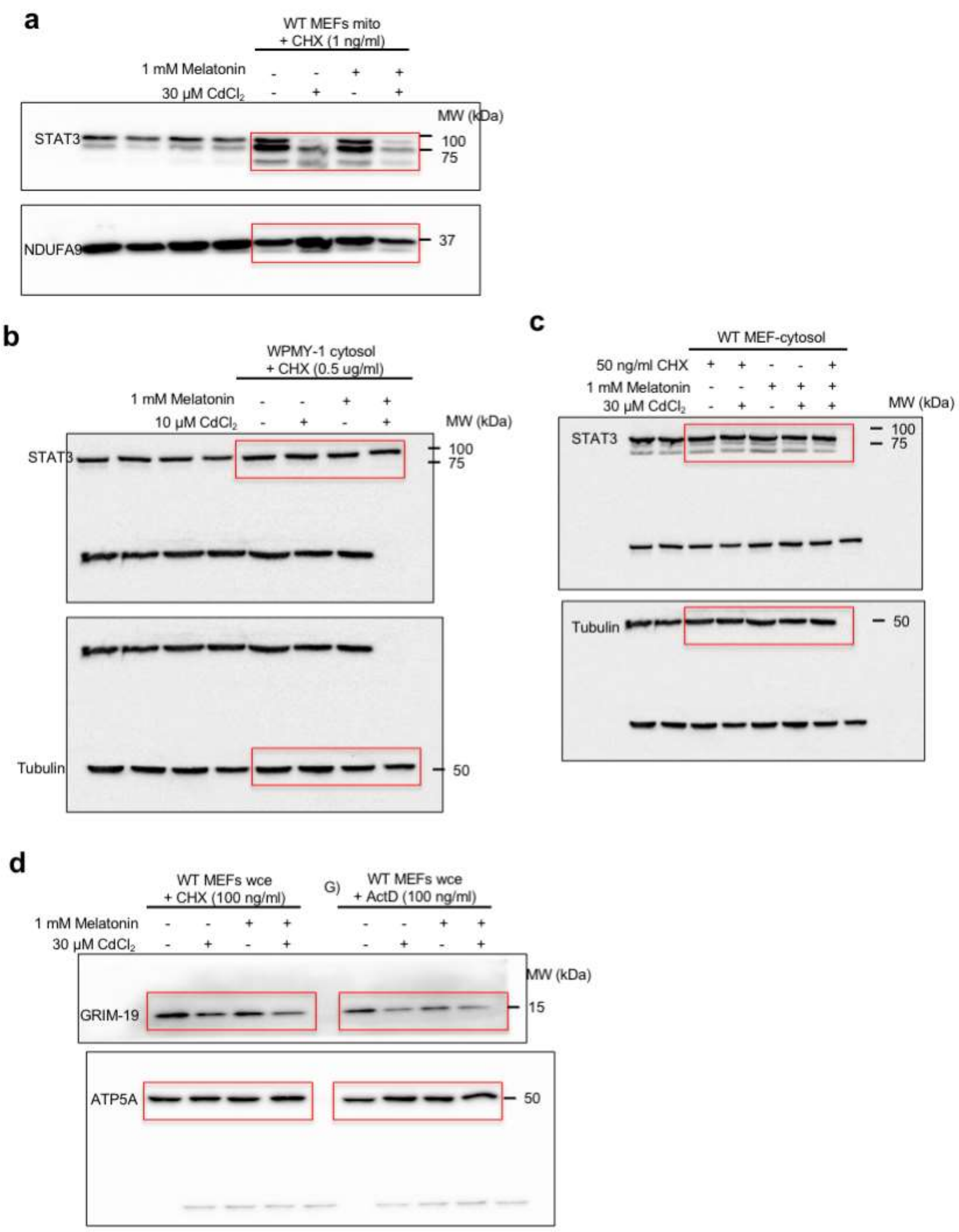

Related to supplementary data figure 5

c

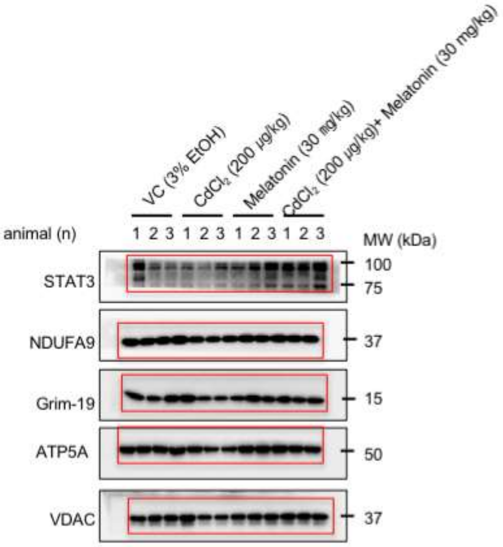

d

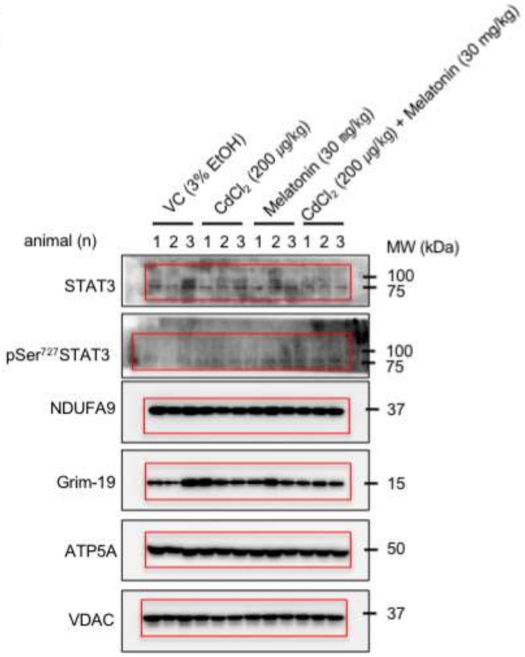

Supplementary Figure 6. Western blot images for figures. Red rectangles indicate cropped representative bands.

WPMY-1 sictrl
untreated

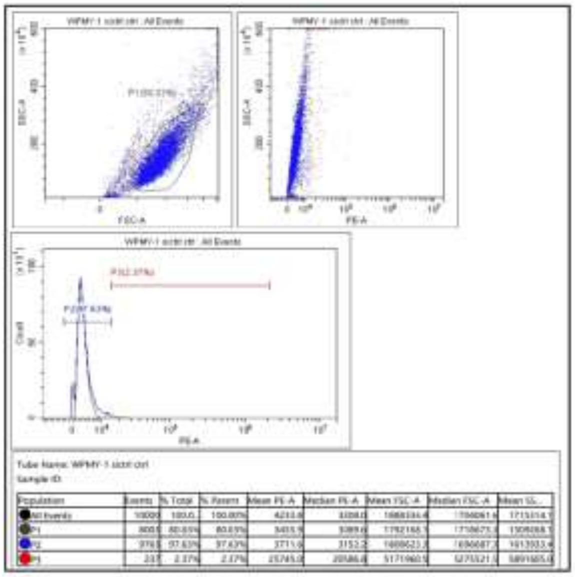

WPMY-1 sictrl
10 µM $CdCl_2$

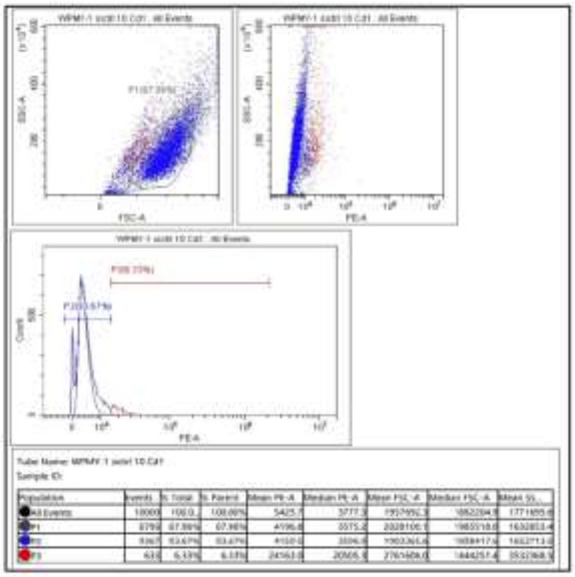

WPMY-1 sictrl
1 mM Melatonin

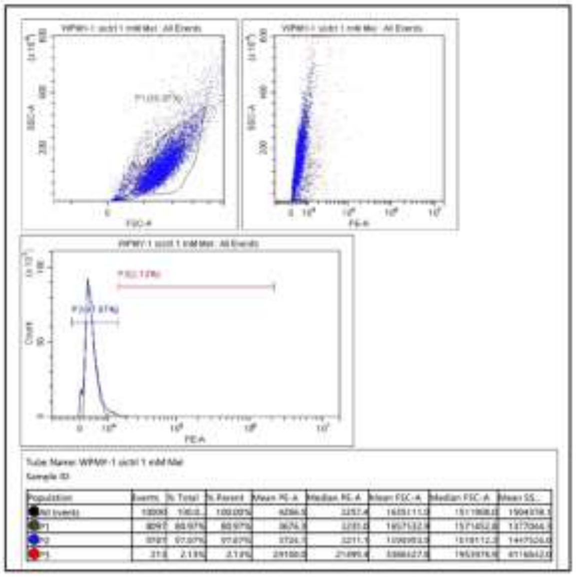

WPMY-1 sictrl
1 mM Melatonin + 10 µM $CdCl_2$

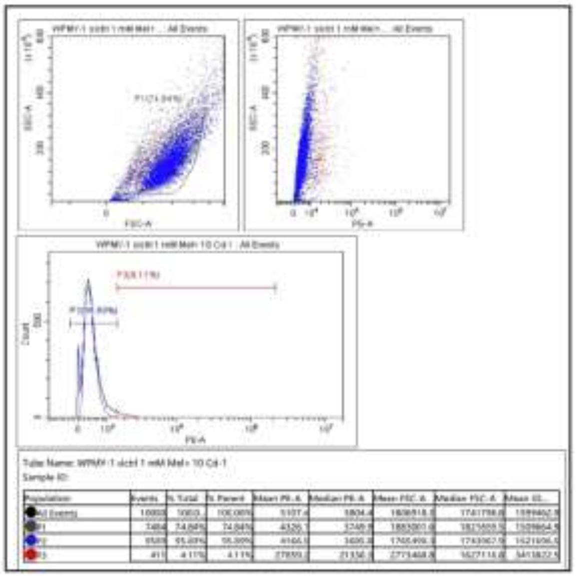

WPMY-1 siSTAT3
untreated

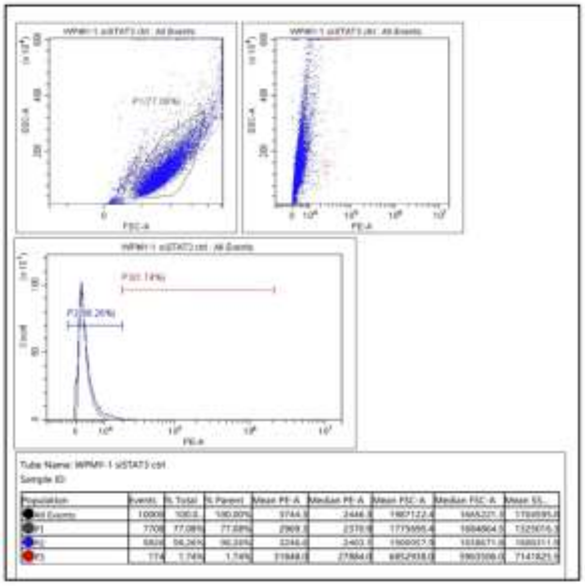

WPMY-1 siSTAT3
10 µM $CdCl_2$

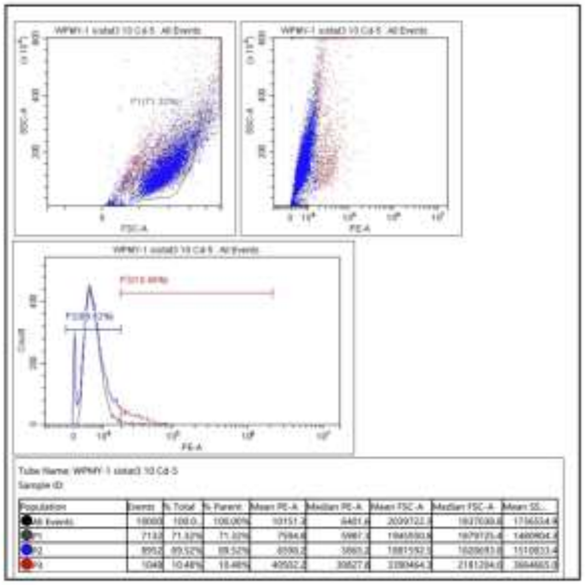

WPMY-1 siSTAT3
1 mM Melatonin

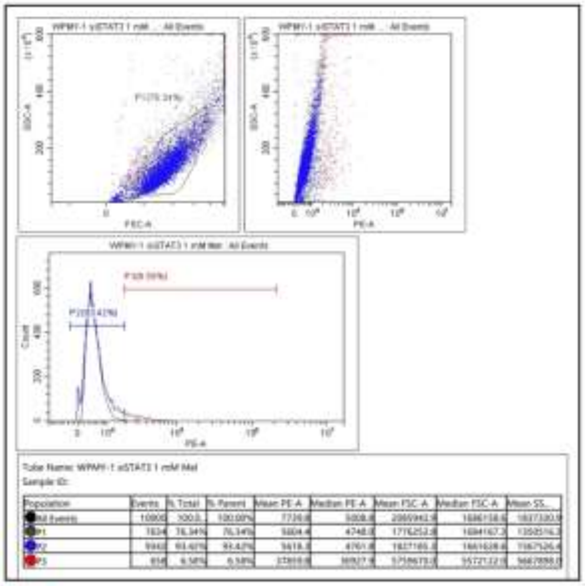

WPMY-1 siSTAT3
1 mM Melatonin + 10 µM $CdCl_2$

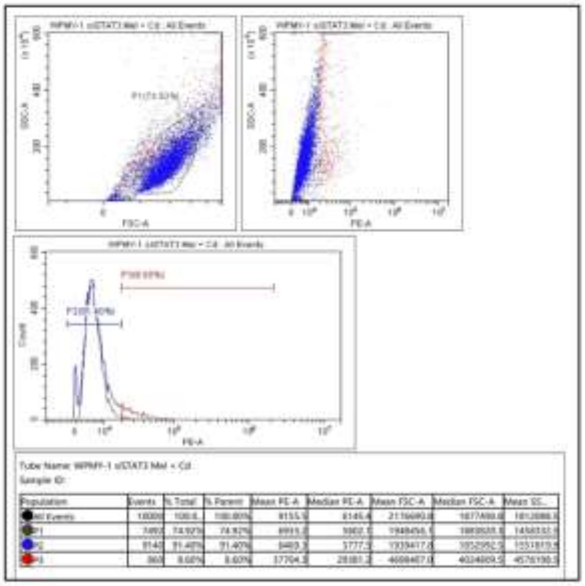

WT MEF
untreated

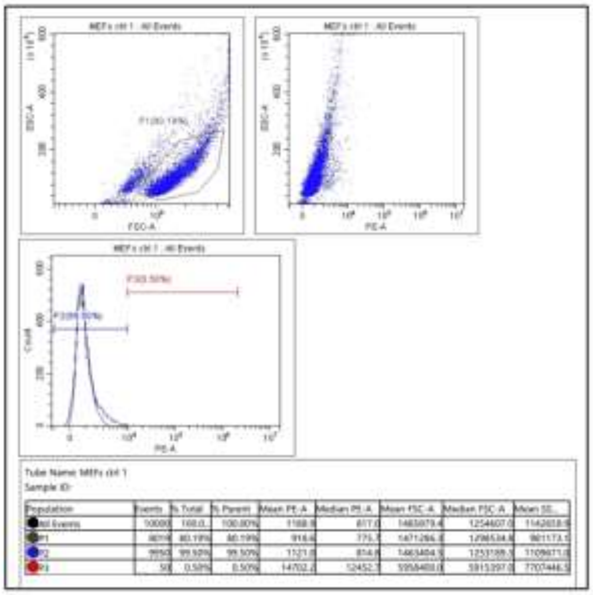

WT MEF
30 µM $CdCl_2$

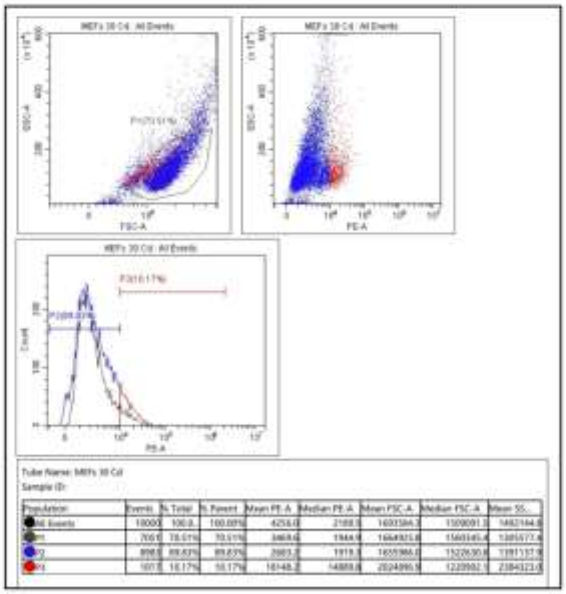

WT MEF
1 mM Melatonin

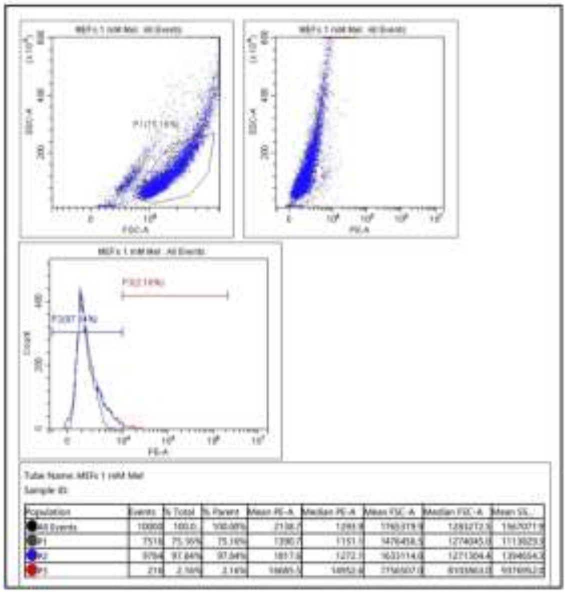

WT MEF
1 mM Melatonin + 30 µM $CdCl_2$

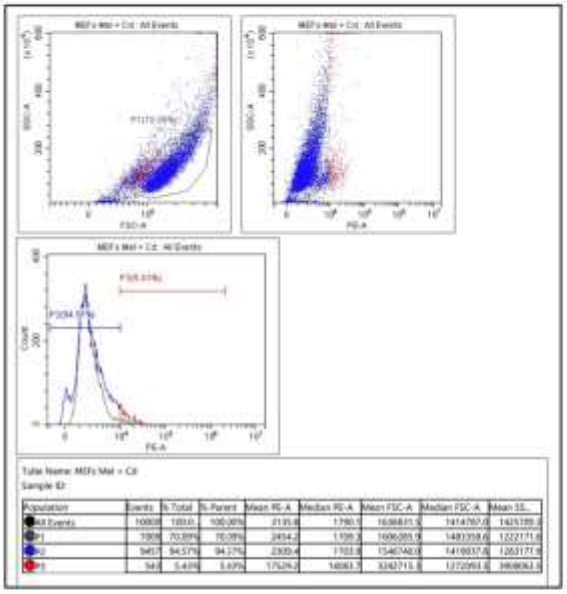

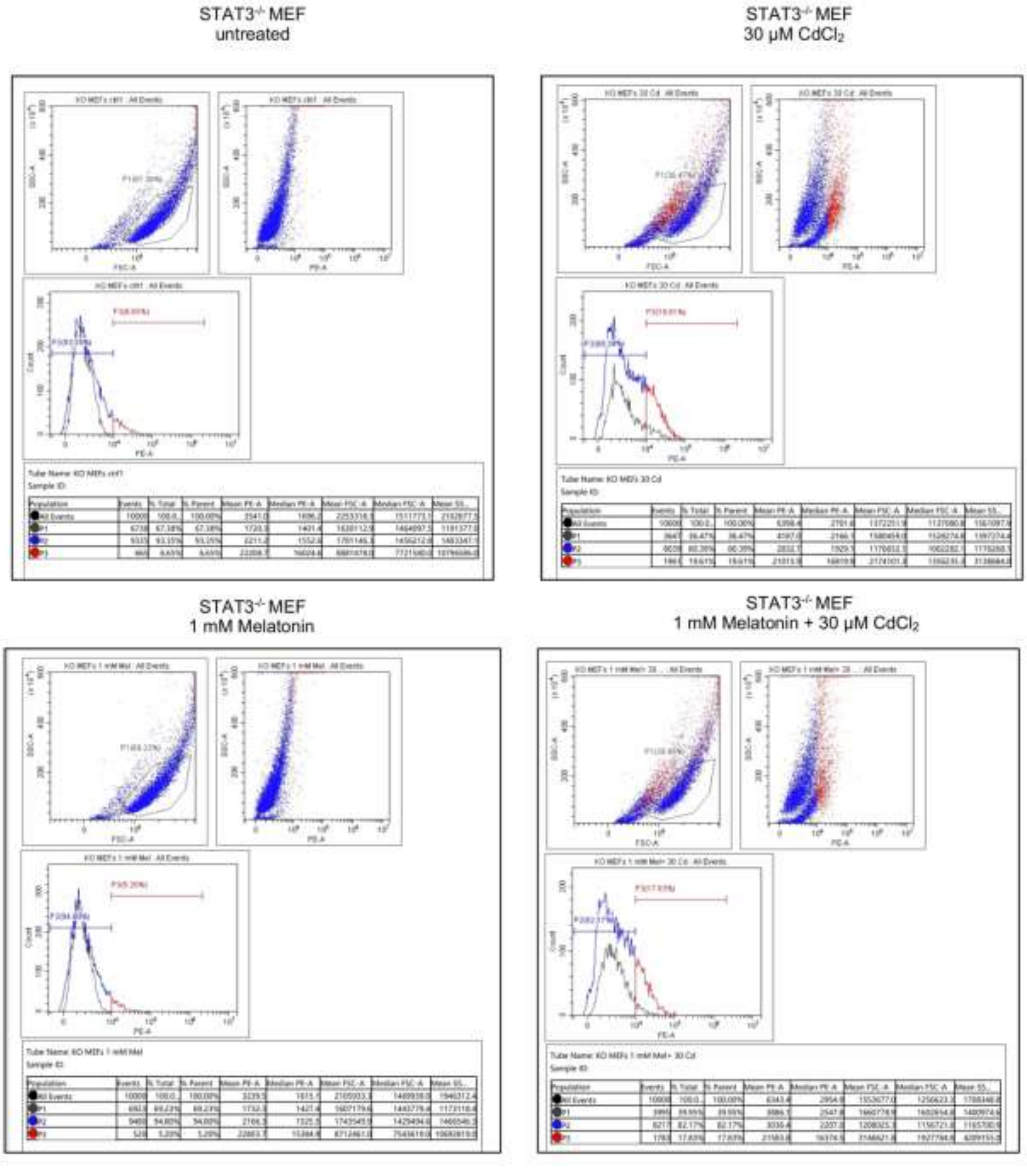

Supplementary Figure 7. Flow cytometry gating strategy for mitoROS staining intensity in figure 3c, d.

WPMY-1 sictrl
untreated

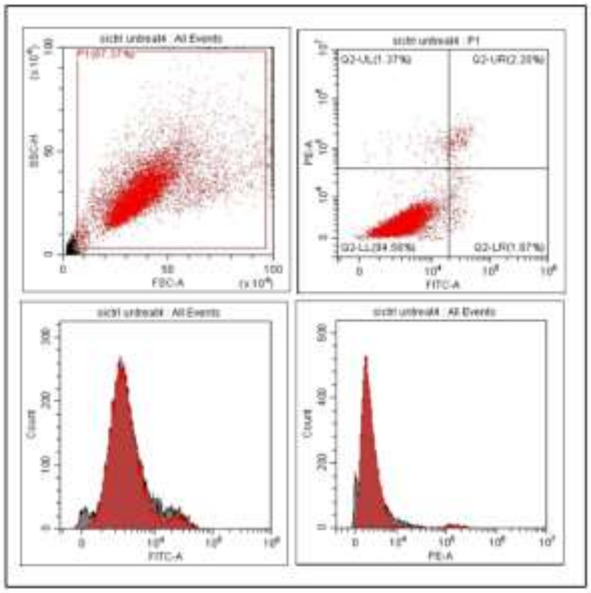

WPMY-1 sictrl
10 µM $CdCl_2$

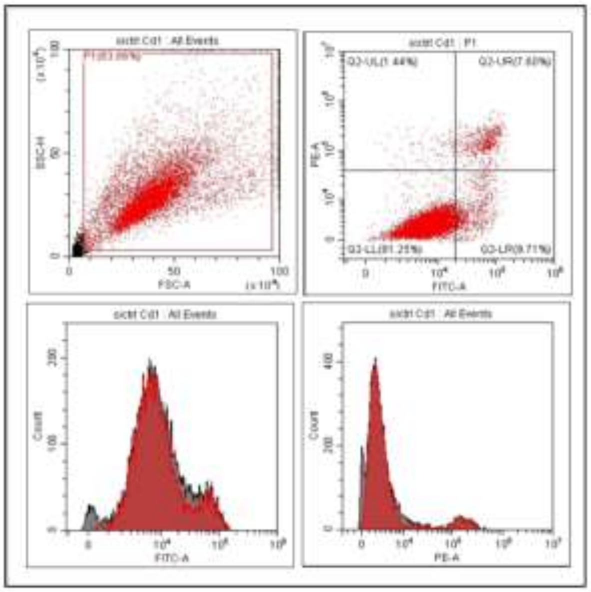

WPMY-1 sictrl
1 mM Melatonin

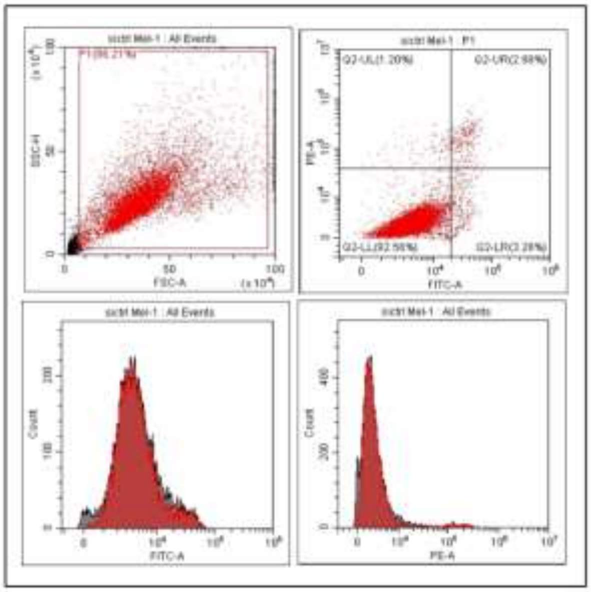

WPMY-1 sictrl
1 mM Melatonin + 10 µM $CdCl_2$

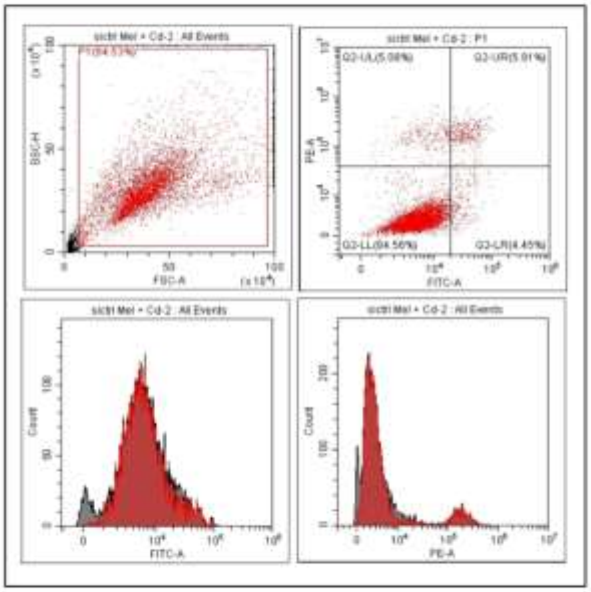

WPMY-1 siSTAT3
untreated

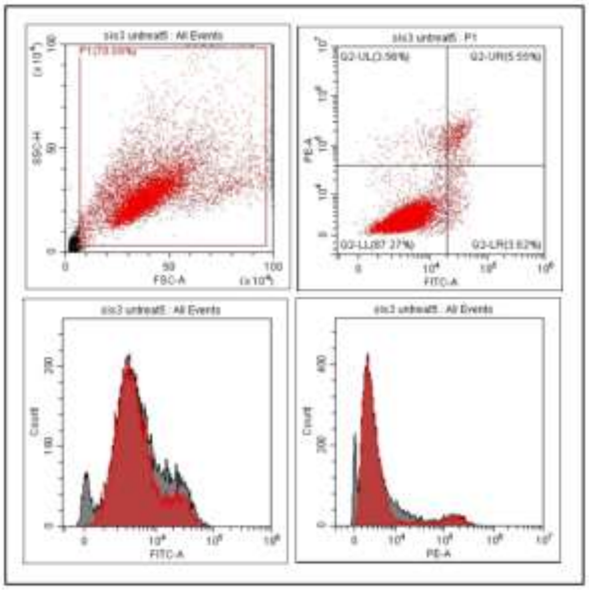

WPMY-1 siSTAT3
10 µM $CdCl_2$

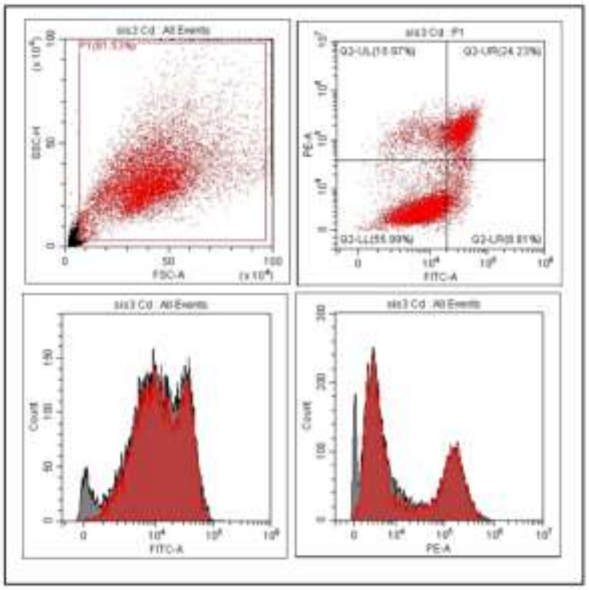

WPMY-1 siSTAT3
1 mM Melatonin

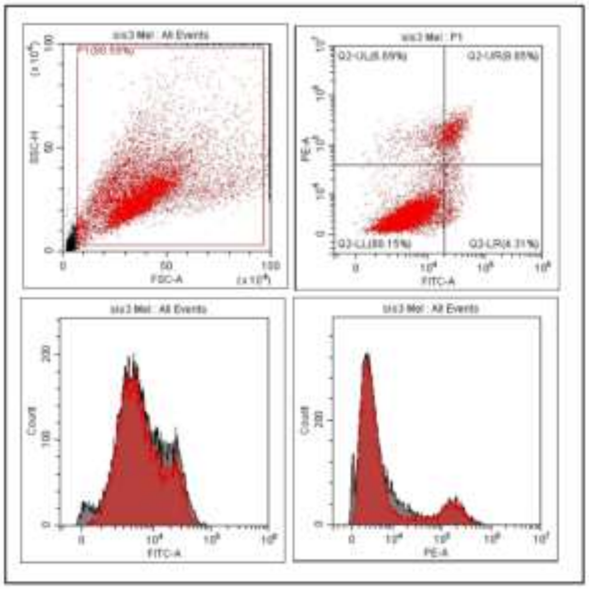

WPMY-1 siSTAT3
1 mM Melatonin + 10 µM $CdCl_2$

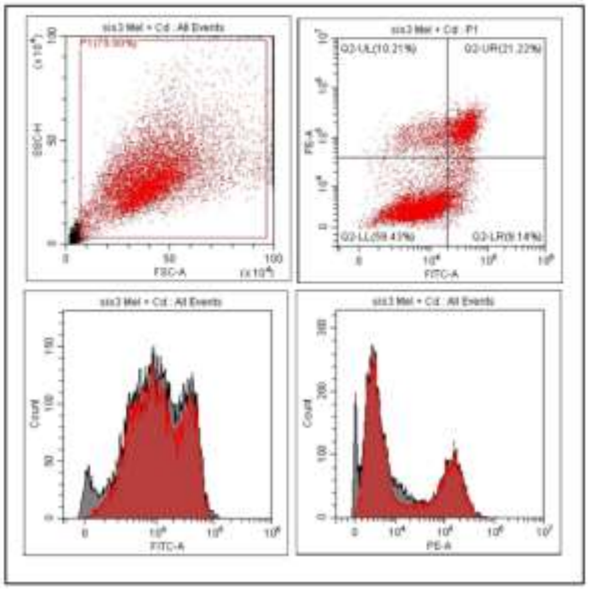

WT MEF
untreated

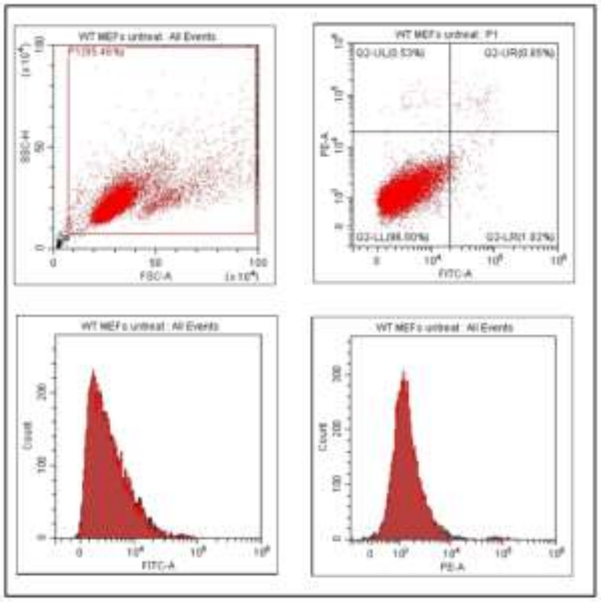

WT MEF
30 µM $CdCl_2$

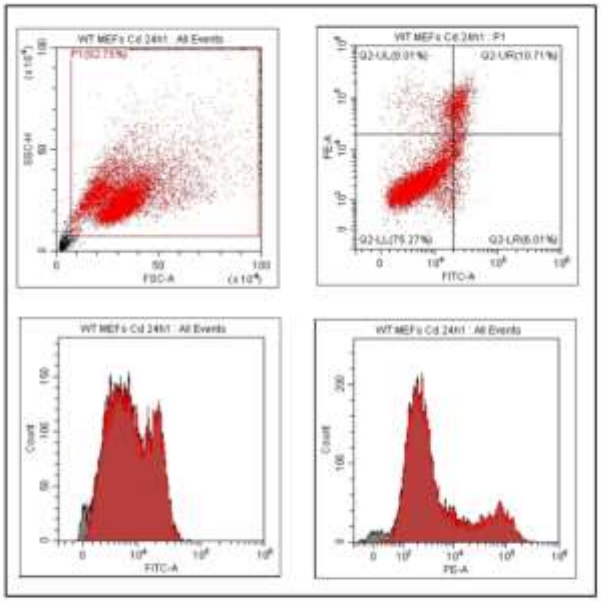

WT MEF
1 mM Melatonin

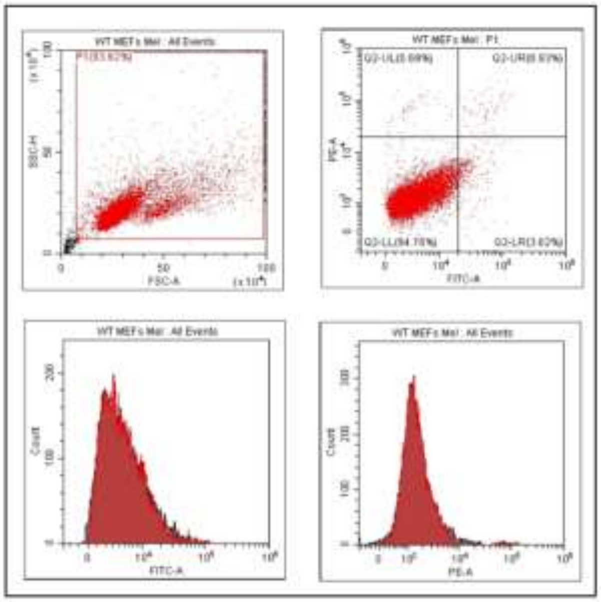

WT MEF
1 mM Melatonin + 30 µM $CdCl_2$

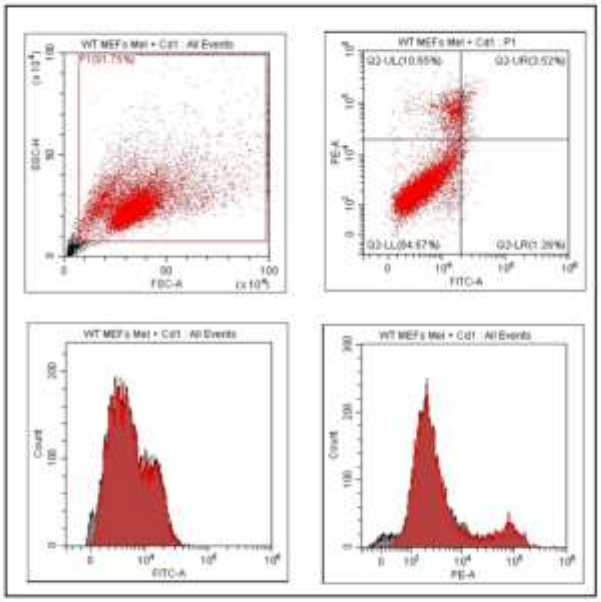

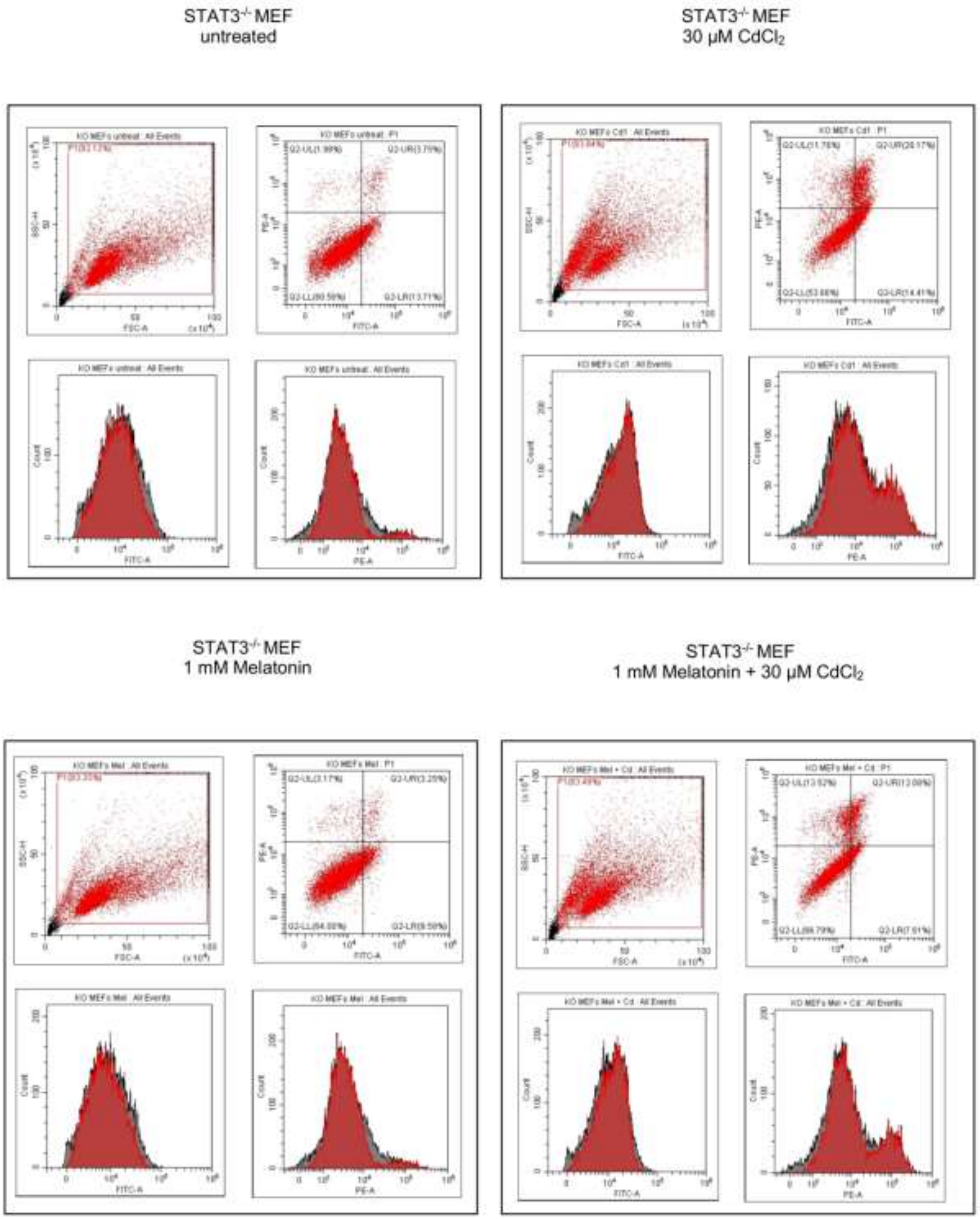

Supplementary Figure 8. Flow cytometry gating strategy for apoptosis assay in the supplementary data figure 1a, b.

WPMY-1 sictrl
untreated

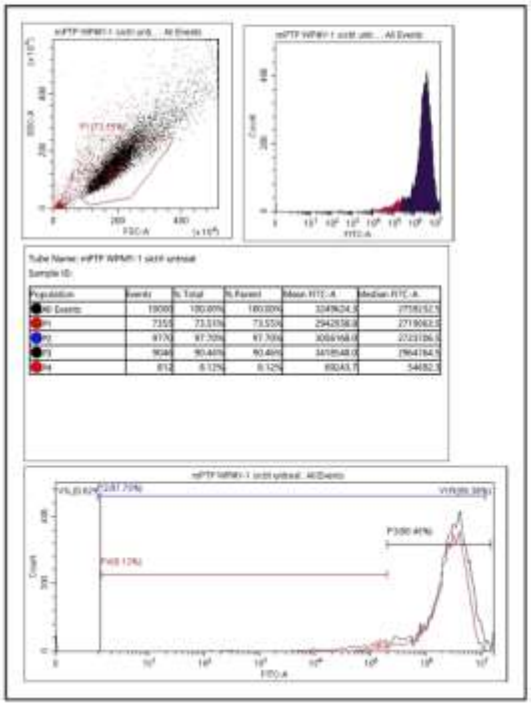

WPMY-1 sictrl
10 µM $CdCl_2$

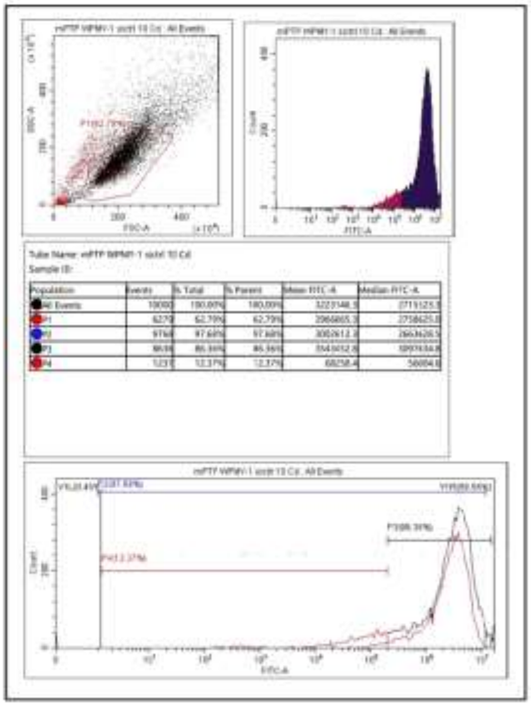

WPMY-1 sictrl
1 mM Melatonin

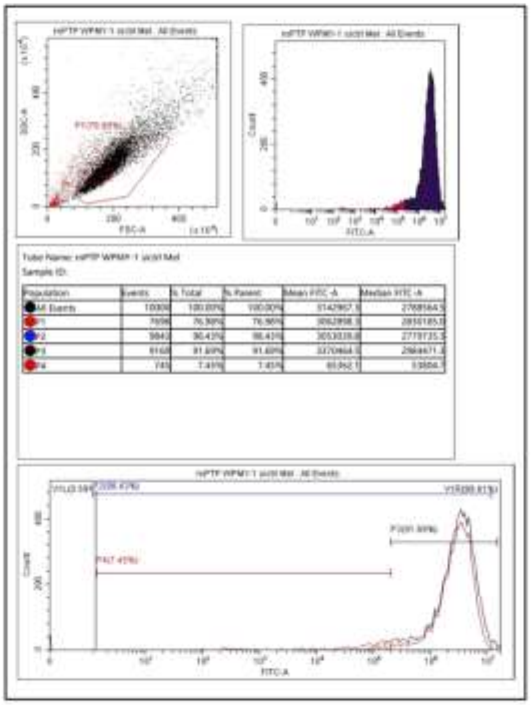

WPMY-1 sictrl
1 mM Melatonin + 10 µM $CdCl_2$

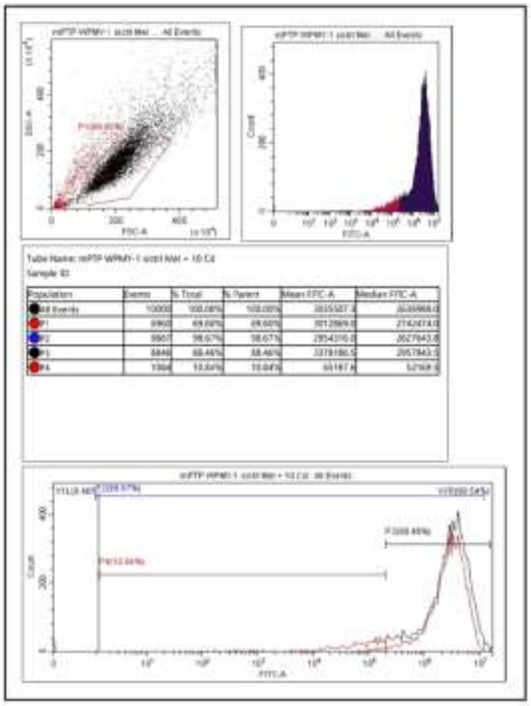

WPMY-1 siSTAT3
untreated

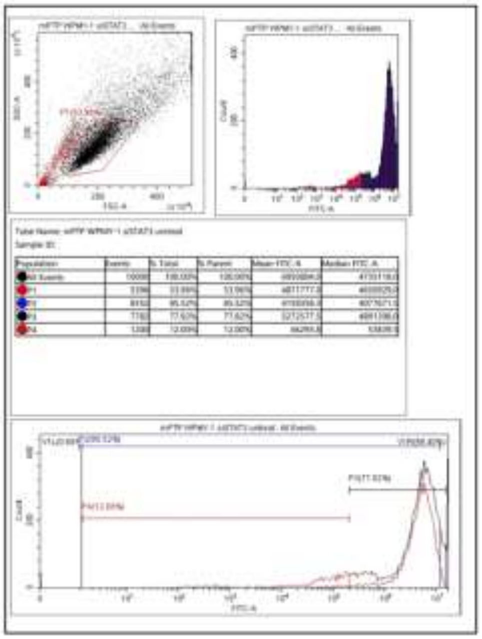

WPMY-1 siSTAT3
10 µM $CdCl_2$

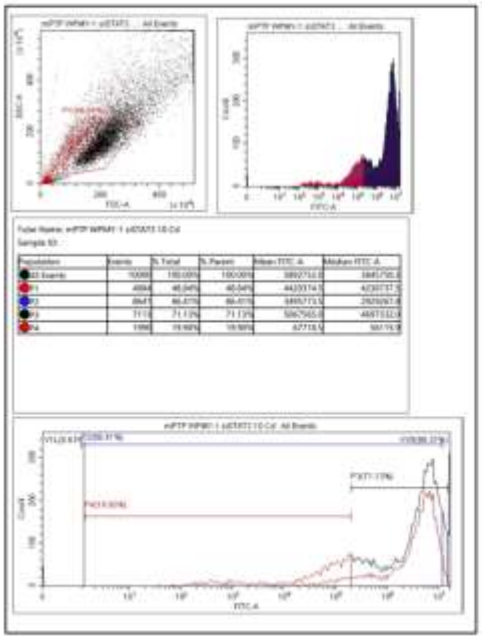

WPMY-1 siSTAT3
1 mM Melatonin

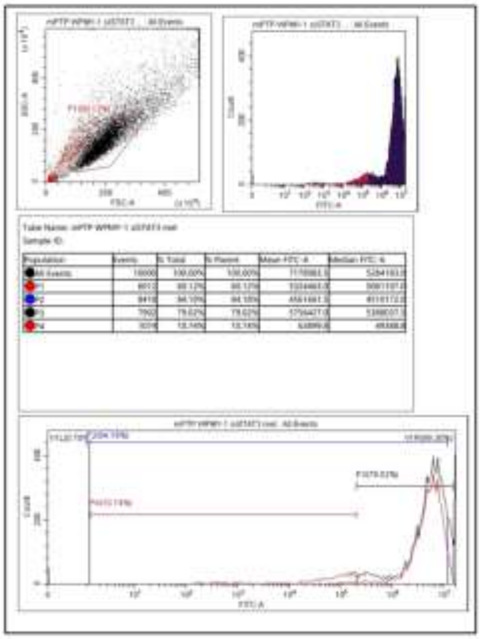

WPMY-1 siSTAT3
1 mM Melatonin + 10 µM $CdCl_2$

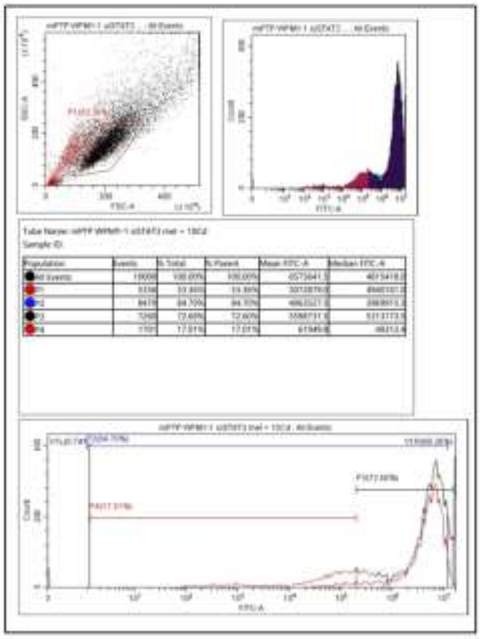

WT MEF
untreated

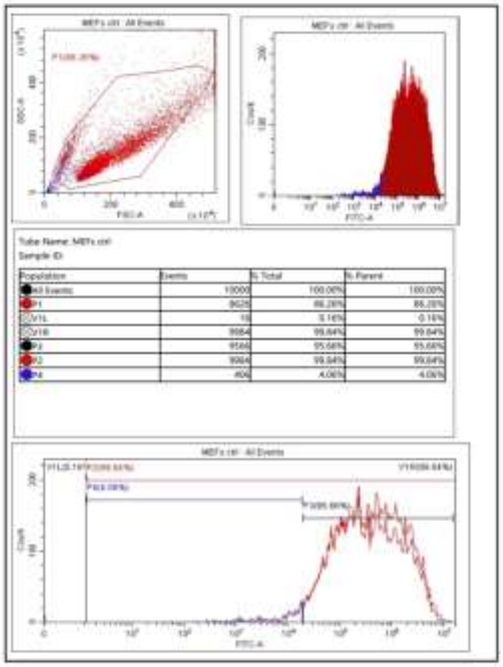

WT MEF
30 μM $CdCl_2$

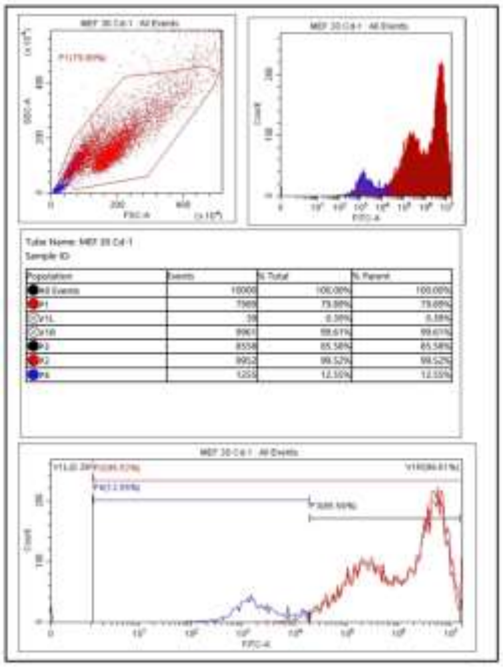

WT MEF
1 mM Melatonin

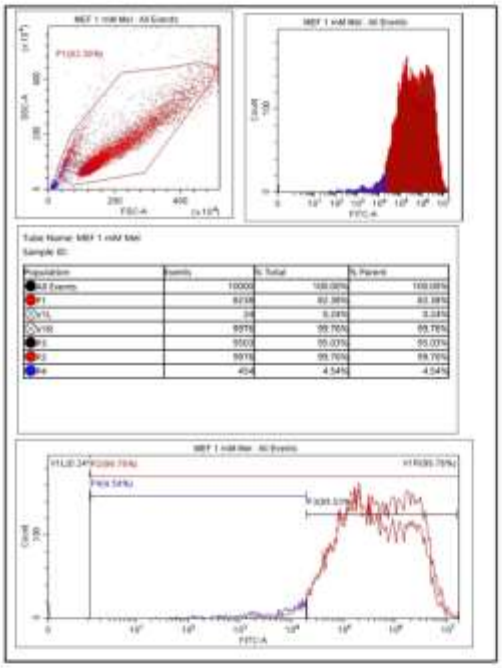

WT MEF
1 mM Melatonin + 30 μM $CdCl_2$

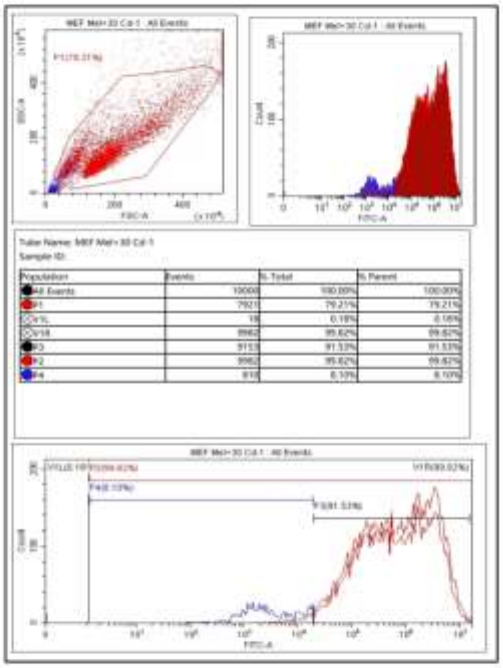

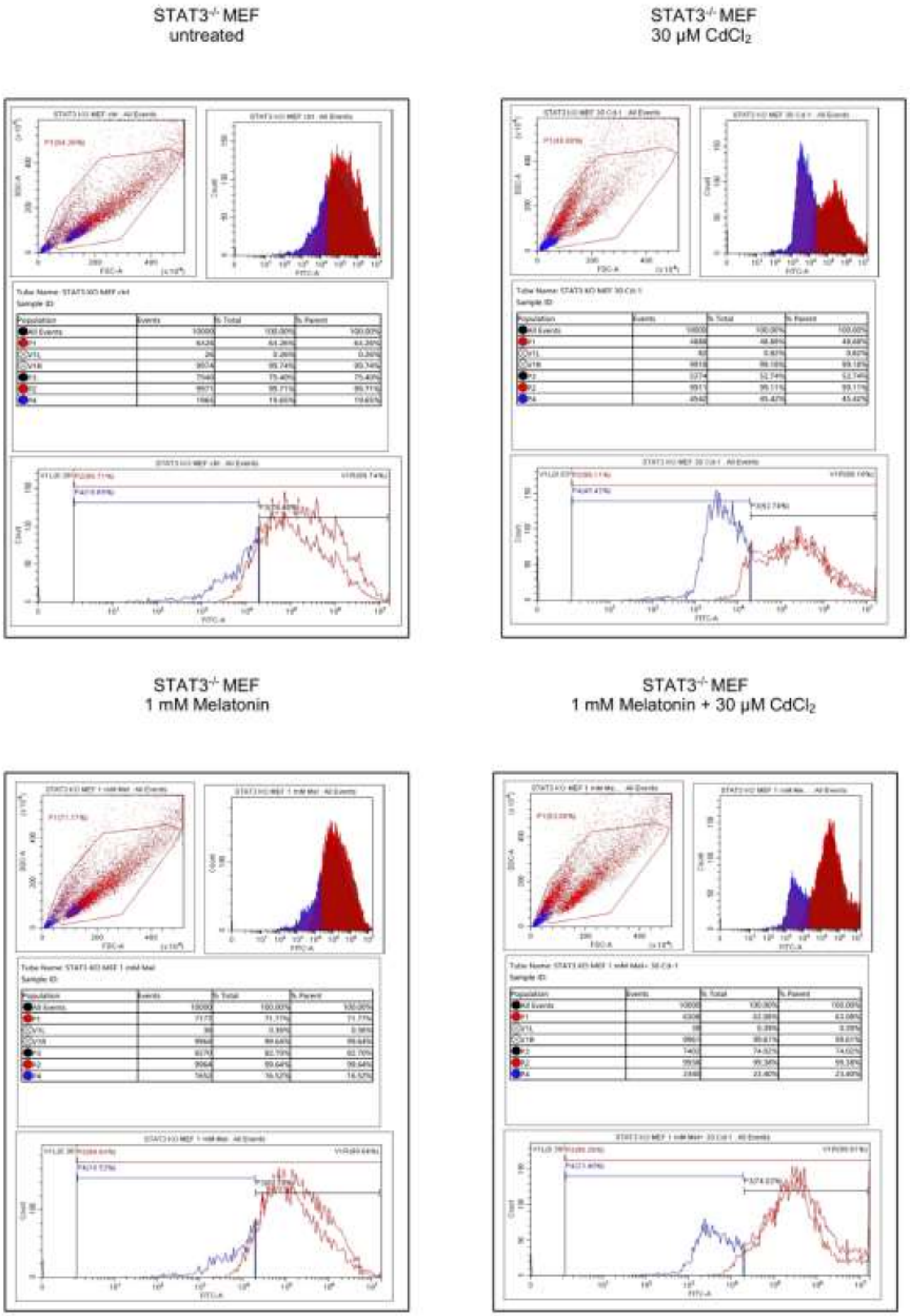

Supplementary Figure 9. Flow cytometry gating strategy for calcein-AM assay in figure 3e, f.
