# Supplementary material for: Melatonin protects against cadmium-induced oxidative stress via mitochondrial STAT3 signaling in human prostate stromal cells
Source: Commun Biol. 2023 Feb 8;6:157. doi: 10.1038/s42003-023-04533-7 (PMC9905543; doi:10.1038/s42003-023-04533-7)
Supplement: Supplementary file 2 — Description of Additional Supplementary Files [file 42003_2023_4533_MOESM2_ESM.pdf]

Description of Additional Supplementary Files

File name: Supplementary Data 1
Description: The numerical source data for graphs.
