# Supplementary material for: Melatonin protects against cadmium-induced oxidative stress via mitochondrial STAT3 signaling in human prostate stromal cells
Source: Commun Biol. 2023 Feb 8;6:157. doi: 10.1038/s42003-023-04533-7 (PMC9905543; doi:10.1038/s42003-023-04533-7)
Supplement: Supplementary file 4 — Reporting Summary [file 42003_2023_4533_MOESM4_ESM.pdf]

nature portfolio

Corresponding author(s): Sung Min Han and Jeong-Doo Heo

Last updated by author(s): 01/23/2023

Reporting Summary

Nature Portfolio wishes to improve the reproducibility of the work that we publish. This form provides structure for consistency and transparency in reporting. For further information on Nature Portfolio policies, see our Editorial Policies and the Editorial Policy Checklist.

Statistics

For all statistical analyses, confirm that the following items are present in the figure legend, table legend, main text, or Methods section.

n/a	Confirmed	
☐	☒	The exact sample size (*n*) for each experimental group/condition, given as a discrete number and unit of measurement
☐	☒	A statement on whether measurements were taken from distinct samples or whether the same sample was measured repeatedly
☐	☒	The statistical test(s) used AND whether they are one- or two-sided<br>*Only common tests should be described solely by name; describe more complex techniques in the Methods section.*
☐	☒	A description of all covariates tested
☐	☒	A description of any assumptions or corrections, such as tests of normality and adjustment for multiple comparisons
☐	☒	A full description of the statistical parameters including central tendency (e.g. means) or other basic estimates (e.g. regression coefficient) AND variation (e.g. standard deviation) or associated estimates of uncertainty (e.g. confidence intervals)
☐	☒	For null hypothesis testing, the test statistic (e.g. *F*, *t*, *r*) with confidence intervals, effect sizes, degrees of freedom and *P* value noted<br>*Give P values as exact values whenever suitable.*
☒	☐	For Bayesian analysis, information on the choice of priors and Markov chain Monte Carlo settings
☒	☐	For hierarchical and complex designs, identification of the appropriate level for tests and full reporting of outcomes
☒	☐	Estimates of effect sizes (e.g. Cohen's *d*, Pearson's *r*), indicating how they were calculated

Our web collection on statistics for biologists contains articles on many of the points above.

Software and code

Policy information about availability of computer code

Data collection	*Provide a description of all commercial, open source and custom code used to collect the data in this study, specifying the version used OR state that no software was used.*
Data analysis	Graphpad Prism Ver. 9.0

For manuscripts utilizing custom algorithms or software that are central to the research but not yet described in published literature, software must be made available to editors and reviewers. We strongly encourage code deposition in a community repository (e.g. GitHub). See the Nature Portfolio guidelines for submitting code & software for further information.

Data

Policy information about availability of data

All manuscripts must include a data availability statement. This statement should provide the following information, where applicable:

- Accession codes, unique identifiers, or web links for publicly available datasets
- A description of any restrictions on data availability
- For clinical datasets or third party data, please ensure that the statement adheres to our policy

All other data supporting the findings of this study are available from the corresponding author upon reasonable request.

Human research participants

Policy information about studies involving human research participants and Sex and Gender in Research.

Reporting on sex and gender	*Use the terms sex (biological attribute) and gender (shaped by social and cultural circumstances) carefully in order to avoid confusing both terms. Indicate if findings apply to only one sex or gender; describe whether sex and gender were considered in study design whether sex and/or gender was determined based on self-reporting or assigned and methods used. Provide in the source data disaggregated sex and gender data where this information has been collected, and consent has been obtained for sharing of individual-level data; provide overall numbers in this Reporting Summary. Please state if this information has not been collected. Report sex- and gender-based analyses where performed, justify reasons for lack of sex- and gender-based analysis.*
Population characteristics	*Describe the covariate-relevant population characteristics of the human research participants (e.g. age, genotypic information, past and current diagnosis and treatment categories). If you filled out the behavioural & social sciences study design questions and have nothing to add here, write "See above."*
Recruitment	*Describe how participants were recruited. Outline any potential self-selection bias or other biases that may be present and how these are likely to impact results.*
Ethics oversight	*Identify the organization(s) that approved the study protocol.*

Note that full information on the approval of the study protocol must also be provided in the manuscript.

Field-specific reporting

Please select the one below that is the best fit for your research. If you are not sure, read the appropriate sections before making your selection.

☒ Life sciences ☐ Behavioural & social sciences ☐ Ecological, evolutionary & environmental sciences

For a reference copy of the document with all sections, see nature.com/documents/nr-reporting-summary-flat.pdf

Life sciences study design

All studies must disclose on these points even when the disclosure is negative.

Sample size	Sample size were determined previous experiments carried out in our laboratories. We perform at least three independent experiments for each assay.
Data exclusions	No data were excluded from the analyses presented in this study.
Replication	Most of the experiments were repeated at least three or five times or triplicate or biological replicates.
Randomization	All chemical treated and untreated control groups were tested. Randomization was not performed as the sample categories for in vitro analysis had to be tracked.
Blinding	Blinding test were not performed as most data are in vitro assays. Some experiments were repeated and confirmed by two independent researchers.

Reporting for specific materials, systems and methods

We require information from authors about some types of materials, experimental systems and methods used in many studies. Here, indicate whether each material, system or method listed is relevant to your study. If you are not sure if a list item applies to your research, read the appropriate section before selecting a response.

Materials & experimental systems

n/a	Involved in the study	
☐	☒	Antibodies
☐	☒	Eukaryotic cell lines
☒	☐	Palaeontology and archaeology
☐	☒	Animals and other organisms
☒	☐	Clinical data
☒	☐	Dual use research of concern

Methods

n/a	Involved in the study	
☒	☐	ChIP-seq
☐	☒	Flow cytometry
☒	☐	MRI-based neuroimaging

Antibodies

Antibodies used	Primary antibodies:<br>anti-STAT3, anti-pTyr705 STAT3, anti-pSer727 STAT3, anti-CypD, anti-ATP5A, anti-NDUFA9, anti-VDAC, anti-Grim-19, anti-tubulin,<br>Secondary antibodies:<br>Anti-mouse IgG peroxidase, anti-rabbit IgG peroxidase
Validation	All antibodies used were validated by the respective commercial source for the application used in this manuscript.

Eukaryotic cell lines

Policy information about cell lines and Sex and Gender in Research

Cell line source(s)	MEFs were provided by Dr. Andrew Larner (Virginia Commonwealth University, Virginia, U.S.A).<br>WPMY-1 cells were purchased from the ATCC (#CRL-2854).
Authentication	non of the used cell lines were authentication
Mycoplasma contamination	the cell lines were not tested for mycoplasma contamination
Commonly misidentified lines<br>(See ICLAC register)	*Name any commonly misidentified cell lines used in the study and provide a rationale for their use.*

Animals and other research organisms

Policy information about studies involving animals; ARRIVE guidelines recommended for reporting animal research, and Sex and Gender in Research

Laboratory animals	Six-week-old male CD-1 (Institute for Cancer Research; ICR) mice weighing 20–22 g were purchased from Orient Bio Company (Seongnam, Korea).
Wild animals	This manuscript does not involve wild animals.
Reporting on sex	We used male mice and described in the manuscript.
Field-collected samples	This study does not involve samples collect from the field.
Ethics oversight	All experiments were approved by the Institutional Agricultural Animal Care and Use Committee of KIT (IACUC No.2011-0005)

Note that full information on the approval of the study protocol must also be provided in the manuscript.

Flow Cytometry

Plots

Confirm that:

- [x] The axis labels state the marker and fluorochrome used (e.g. CD4-FITC).
- [x] The axis scales are clearly visible. Include numbers along axes only for bottom left plot of group (a 'group' is an analysis of identical markers).
- [x] All plots are contour plots with outliers or pseudocolor plots.
- [x] A numerical value for number of cells or percentage (with statistics) is provided.

Methodology

Sample preparation	Apoptosis: WPMY-1 and MEFs cells were evaluated by staining with Annexin V and with propidium iodide to identify dead cells. Harvest the cells were resuspended in 1x annexin-binding buffer with 5 uL of annexin v and 1 uL of PI. Cells were incubated at room temperature for 15 min. After washing with PBS three times, the cells were resuspended in 1x annexin binding buffer for analysis.<br>MitoROS: Harvest the cells were resuspended in 1x HBSS with 5 uM MitoSOX reagent. Cells were incubated at 37 ? for 15 min. After washing with PBS three times, the cells were resuspended in 1x PBS for analysis.<br>mPTP: Harvest the cells were resuspended in 1x HBSS with each reagent. Tube 1 containing only calcein AM. Tube 2 containing calcein AM and CoCl2. Tube 3 containing calcein AM, CoCl2 and Ionomycin. Samples were prepared respectively. Cells were incubated at 37 ? for 15 min. After washing with PBS three times, the cells were resuspended in 1x PBS for analysis.
Instrument	CytoFLEX (BECKMAN COUTER)
Software	CyExpert

Cell population abundance | Almost 100% of the sample are shown.

Gating strategy | Apoptosis: Live cell population was gated on FSC vs SSC plot, or propidium iodide negative population.
mitoROS: Live cell population was gated on FSC vs SSC plot, or mitoROS red positive population.
mPTP: Live cell population was gated on FSC vs SSC plot. Analyze the samples using a flow cytometer with 488 nm excitation and emission filter for fluorescein (Molecular Probes, #M34153).

☐ Tick this box to confirm that a figure exemplifying the gating strategy is provided in the Supplementary Information.
